# Schisandrin B Targets PXR to Enhance Bile Acid Metabolism and Alleviate ANIT-Induced Cholestatic Liver Injury via Dual Pathways

**DOI:** 10.7150/ijbs.121475

**Published:** 2026-01-01

**Authors:** Ying Zhang, Huan Lan, Xuechun Yu, Lin Zhuo, Bixin Zhao, Fang Liu, Lin An, Fan Zhang, Zhongqiu Liu, Caiyan Wang

**Affiliations:** 1 State Key Laboratory of Traditional Chinese Medicine Syndrome, International Institute for Translational Chinese Medicine, Guangzhou University of Chinese Medicine, Guangzhou, Guangdong, China.; 2 Chinese Medicine Guangdong Laboratory (Hengqin Laboratory), Guangdong-Macao In-Depth Cooperation Zone in Hengqin, Zhuhai 519000, China.

**Keywords:** Schisandrin B, PXR, bile acid metabolism, TFEB, cholestatic liver injury

## Abstract

Cholestatic liver injury (CLI) is a rapid progressive liver disorder characterized by the accumulation of bile acids (BA). Although pregnane X receptor (PXR) is a critical regulator of BA metabolism, the synergistic mechanisms of natural compounds targeting these pathways remain unclear. In this study, we demonstrated a positive correlation between BA accumulation and disease severity in clinical samples. Further, we identified Schisandrin B (Sin B), a lignan from *Schisandra chinensis*, as a potent hepatoprotective agent in α-naphthyl isothiocyanate (ANIT)- induced CLI. We demonstrated that Sin B treatment reduced BA levels and inflammation in ANIT-induced WRL68 cells, liver lobule chips, and mice. Notably, Sin B activated PXR, increased the levels of UDP-glucuronosyltransferase 1A1 (UGT1A1), CYP3A4 (in humans) / CYP3A11 (in mice) and MRPs, and enhanced TFEB transcriptional activity and autophagic flux *in vivo* and* in vitro*. Knockout of hepatic *Pxr* or *Tfeb* blocked these effects of Sin B. Mechanistic investigation revealed that Sin B is directly binds to PXR at residues S106, G144, and W299, inducing conformational changes in the ligand-binding domain (LBD) was verified through target fishing, molecular dynamics (MD) simulations, drug affinity responsive target stability assay, isothermal titration calorimetry and surface plasmon resonance. Our findings provide structural and functional insights into the dual-pathway mechanism of Sin B and support its therapeutic potential for CLI.

## 1. Introduction

Cholestatic liver injury (CLI) is characterized by bile acid (BA) accumulation, bile duct destruction, and inflammation, which together lead to hepatocyte damage 1. Obeticholic acid (OCA) and ursodeoxycholic acid (UDCA) are often used to treat CLI 2-4. They regulate BA synthesis and metabolism by activating members of the nuclear receptor family 5, 6. However, they exhibit limited efficacy and cause adverse effects such as pruritus and diarrhea in approximately 46% of patients 7. Therefore, new therapeutic drugs and strategies for treating CLI are urgently required.

Pregnane xenobiotic receptor (PXR) is an important nuclear receptor that regulates the expression of cytochrome p450 enzymes (CYPs) and BA transporters, promotes bile secretion in hepatocytes, supports BA metabolism *in vivo*, and fosters anti-CLI effects 8-11. The N-terminal region of the PXR contains a DNA-binding domain that binds to response elements in the promoter regions of target genes and the C-terminal region comprises the ligand-binding domain (LBD) which contains a depression that interacts with its ligand as well as with activation function 2 (AF-2) 12. Studies have shown Pregnane 16a-carbonitrile (PCN), a PXR agonist in rodents, significantly improves lithocholic acid (LCA) -induced cholestasis 13. Consistent with this, clinical studies have shown that rifampicin, an active PXR ligand in humans, upregulates PXR activation-mediated phase II UDP-glucuronosyltransferase 1A1 (UGT1A1) and resistance-associated protein 2 (MRP2), contributing to therapeutic effects on cholestasis 14. In addition, transcription factor EB (TFEB), a central regulator of the autophagolysosomal signaling pathway, is involved in BA and lipid metabolisms 15, 16. In mice fed a high-fat diet, continuous inhibition of BA uptake by the sodium BA transporter enhances hepatic TFEB nuclear localization and improves BA homeostasis 15. Further, TFEB was found to control the transcriptional activation of the lipid-degrading cell modulator PGC1α and regulate the expression of CYP7A1 to modulate liver BA synthesis 17, 18. Studies have shown that activation of PXR regulates TFEB expression. Thus, manipulation of the PXR-TFEB signaling pathway may be a promising therapeutic strategy for CLI. However, the specific structural basis of ligand binding by PXR remains unclear, limiting the development of nuclear receptor-targeting drugs.

As a traditional Chinese herbal medicine, *Schisandra chinensis* has been historically used in clinical practice. The main active components of *Schisandra chinensis* are the lignans schisandrin A (Sin A), schisandrin B (Sin B), schisandrol A (Sol A), schisandrol B (Sol B), schisanhenol (Son L), schisantherin A (Shin A), and schisantherin B (Shin B). Modern pharmacological studies have shown that these ingredients play important roles in liver protection by reducing alanine aminotransferase (ALT), aspartate aminotransferase (AST) levels and promoting hepatocyte proliferation, especially in LCA-induced CLI 13, 19. Sol B activates foresaid X receptor (FXR), which inhibits the expression of CYP7A1, thereby inhibiting BA synthesis and reducing BA accumulation in the liver 20, 21. Furthermore, Sol B alleviates the liver inflammation by accelerating BA metabolism in the liver and gut 19, 22, 23. Lignans exhibit significant potential for combating liver diseases, and various studies have revealed that they act on different targets to promote BA metabolism. However, systematic comparative studies evaluating the specific effects of different lignan components on CLI are lacking. Additionally, the mechanism by which these components synergistically interact with distinct targets at the structural biology level to enhance BA metabolism remains unresolved.

In this study, we found a positive correlation between BA accumulation and the severity of CLI. Further, using liver organoid chips and animal models, we show that Sin B from *Schisandra chinensis* shows gallbladder and liver protection. We found that Sin B interacts with key sites of PXR and induces conformational changes in the PXR-LBD domain. Furthermore, we confirmed that Sin B targets PXR to promote the transcription of BA metabolism enzymes and lysosomal autophagy-related genes. These findings highlight the mechanisms underlying the protective effects of Sin B in CLI and provide new potential strategies for the clinical treatment of liver diseases.

## 2. Materials and Methods

### 2.1 Reagents

Alpha-naphthylisothiocyanate (ANIT) was purchased from Sigma (CAS: 551-06-4). Sin A (B21326), Sin B (B21327), Sol A (B21322), Sol B (B21323), Son L (B21324) Shin A (B21328), and Shin B (B21330) were purchased from Yuanye Biotechnology (Chengdu, China). The antibodies β-actin (81115-1-RR, Proteintech, Wuhan, China), γH2AX (AF6187, Affinity Biosciences, Beijing, China), CYP3A11 (18227-1-AP, Proteintech, Wuhan, China), GAPDH (10494-1-AP, Proteintech, Wuhan, China), Histone-H3 (17168-1-AP, Proteintech, Wuhan, China), PXR (67912-1-Ig, Proteintech, Wuhan, China), TFEB (13372-1-AP, Proteintech, Wuhan, China), P62 (66184-1-1g, Proteintech, Wuhan, China), LC3 (18725-1-AP, Proteintech, Wuhan, China), CAR (YP-mAb-13161, UpingBio, Hangzhou, China), FXR (25055-1-AP, Proteintech, Wuhan, China), TGF-β1 (YP-mAb-15967, UpingBio, Hangzhou, China), Collagen I (YP-mAb-137694, UpingBio, Hangzhou, China) and UGT1A1 (23495-1-AP, Proteintech, Wuhan, China) were used for western blotting. LysoTracker Red DND-99 (50 nmol/L, 30 min) was used to stain the lysosome compartments (C1046, Beyotime, Shanghai, China). The antibodies γH2AX and PXR (12869-1-AP, Proteintech, Wuhan, China) were used for immunofluorescence staining.

### 2.2 Collection of human specimens and evaluation of inflammatory levels

Clinical blood samples from eleven patients with liver disease were collected from the First Affiliated Hospital of Guangdong Province; of these samples, eight were from patients with high BA levels and three were from patients with low BA levels. The experimental protocol involving human subjects received approval from the First Affiliated Hospital of Guangdong Province and was conducted under the restriction of the ethical guidelines. Written informed consent was obtained from all participants included in the study. Finally, we centrifuged clinical blood samples at 3,500 rpm for 10 min at 4°C, and the serum was harvested. The content of IL-1β, IL-6 and TNF-α was quantified using ELISA Kit (FineTest Biotech, EH0185, EH0201 and EH0302).

### 2.3 Animal experiments

Male C57BL/6 mice (specific pathogen-free, 6-8 weeks old, body weight 20 ± 2 g) were obtained from the Guangzhou Laboratory Animal Center; the relevant animal production license was SCXK 2022-0002 and laboratory license SYXK 2019-0144. Prior to the experiment, the mice were kept at the Guangzhou University Chinese Medicine Center under a 12 h light/12 h dark cycle with a stable temperature (24 ± 2°C), humidity (50 ± 5%), and an air exchange rate of 10-20 times per hour. Mice had free access to water and SPF mouse feed. After a quarantine, mice were divided into a normal group and the following treatment groups: ANIT model group (40 mg/kg), ANIT+OCA positive group, ANIT+Sin B 25 mg/kg, ANIT+Sin B 50 mg/kg, and ANIT+Sin B 100 mg/kg. There were six mice per group. The ANIT was dissolved in corn oil, and Sin B was dissolved in a 0.5% sodium carboxymethyl cellulose solution (CMC-Na). The different doses of Sin B were administered daily for five consecutive days, and the mice were then dosed with ANIT for two consecutive days starting from day six. Mice were weighed throughout the study and sacrificed 24 hours after the last ANIT treatment for blood collection and subsequent analysis. The liver and other organs were collected, weighed, and photographed. The animal experimental procedures were approved by the Guangzhou University of Chinese Medicine and were following the NIH Guide for the Care and Use of Laboratory Animals (Approval No. SYXK 2024-0144).

### 2.4 Generation of liver-specific *Tfeb* knockout mice

Tfeb Flox/Flox (*Tfeb*^flox/flox^) mice were generated as described previously 24 and were crossed with Albumin Cre (Alb-Cre, C57BL/6J, Jackson Laboratory) for at least nine generations to generate liver-specific *Tfeb* KO (*Tfeb*^flox/flox^, Albumin-Cre+, Liver-*Tfeb* KO) mice. Alb-Cre- (*Tfeb*^flox/flox^, Albumin-Cre-, WT) matched littermates were used in this study. All mice were maintained at 24 ± 2°C and 50 ± 5% humidity. Mice were offered sterile standard normal diet and water ad libitum.

### 2.5 Generation of liver-specific *Pxr* knockdown mice

The shRNA adeno-associated virus package that lowers *Pxr* is provided by Heyuan Biotechnology (Shanghai) Co., LTD. C57BL/6 mice that have passed the 5-day quarantine and weigh about 22 g are prepared to be injected with AAV adeno-associated virus. According to the AAV-PXR shRNA virus with a virus titer of 3.5 × 10^12^ and the AAV-Control empty control, 1 mL of syringe was inhaled and injected into the tail vein of the mice to ensure that all the liquid was pushed into the tail vein. Mice that had been injected with the relevant adenovirus and raised for 4 weeks were dissected to obtain liver tissues. Some of the tissues were frozen and sectioned for tissue immunofluorescence staining. The fluorescence intensity of EGFP carried by AAV9 in the liver tissues was detected to investigate the transfection efficiency of adenovirus. Then, the expression levels of PXR protein and mRNA were determined by western blot and RT-qPCR to further confirm the knockdown efficiency of *Pxr*.

### 2.6 Cell culture and MTT assay

Human Fetal Hepatic Cell Line WRL68 hepatocytes (Shanghai Academy of Biological Sciences, China) were cultured in DMEM supplemented with 10% fetal bovine serum, Shanghai Academy of Biological Sciences, Shanghai Ike Biotechnology Co. The WRL68 cells were cultured with DMEM medium added with 10% fetal bovine serum and 1% penicillin streptomycin at 37°C and 5% CO_2_. To determine cell viability via MTT, WRL68 cells were inoculated into 96-well plates at a density of 4×10^3^/well, treated with different concentrations of ANIT for either 12 or 24 h, and then incubated with 20 μL of 5 mg/mL MTT at 37°C for 4 h. The supernatant was discarded, and 100 μL of DMSO was added to dissolve blue formaldehyde crystals from living cells. Finally, the optical density was measured at 570 nm using a microplate spectrophotometer after oscillating samples for 10 min.

### 2.7 5-Ethynyl-2'-deoxyuridine (EdU) assay

The effects of ANIT and Sin B on cell proliferation were determined using an EdU assay kit. WRL68 cells were first treated with 50 μM of ANIT for 12 h to construct a CLI model, and then treated with 5, 10 and 20 μM Sin B for 24 h. The same volume of DMSO was added to the control group. Finally, the cell number and fluorescence were quantified for each sample with a fluorescence microscope.

### 2.8 Western blotting

Cell samples or Tissue samples were treated with RIPA and then homogenized using a tissue grinder at 60 Hz for 4 min. The total protein content of centrifuged samples was quantified via the bradford method. After an SDS-PAGE electrophoresis to separate samples based on their molecular weight, samples were transferred to a PVDF membrane and treated with 5% BSA for 1 h, incubated with anti-nano antibody at 4 °C overnight, and then washed with TBST three times. Samples were then incubated with enzyme-labeled secondary rabbit or mouse antibodies at room temperature for 1-2 h and then washed with TBST (10 min per wash). Finally, the ECL reagents were used for the exposure, Image J for gray analysis and quantification.

### 2.9 Quantitative real-time PCR analysis (qRT-PCR)

Cell and liver tissue samples were treated with Trizol, and total RNA was extracted using a chloroform-based procedure. The concentration and purity of the extracted total RNA were then measured. Forward and reverse primers for each target gene were added to the samples, along with SYBR Green qPCR Master Mix reagent **(Table 1)**. Finally, samples (collated on a 384-well plate) were analyzed on an ABI7500 instrument to quantify expression of the target genes. With GAPDH as an internal control, relative mRNA levels were determined via the 2^ΔΔ^CT method.

### 2.10 Measurement of alkaline phosphatase (ALP), ALT and AST level

Both cell, clinical blood samples and mouse serum samples were collected after the above-mentioned treatments. The level of AST, ALT, and ALP were determined using the matching kits (C010-2-1, C009-2-1, and A059-2-2 Nanjing Jiancheng, Nanjing, China) in accordance with the manufacturer's instructions*.*

### 2.11 Fabrication of the biomimetic liver lobule chips

To fabricate the mold of the bottom and top layers of the liver lobule chips, lithography technique was used 25. For the top and bottom layers, we prepared polydimethylsiloxane (PDMS, Dow Corning, Michigan, USA), poured it onto the molds and cured it in an oven. After PDMS of the bottom layer was carefully peeled off the wafer, the middle layer was bonded to the bottom layer after oxygen plasma treatment for 1 min; and baked in an oven (2 h at 80 °C). Then, micro-holes of hepatic artery (HA) and central vein (CV) (diameter; 100 μm) and holes of portal vein (PV) and cell-laden extracellular matrix were fabricated on the middle layer using a puncher machine. The liver lobule chips were sterilized using UV light for 60 min prior to use. In the liver, the sum of hepatocytes, hepatic stellate cells, and hepatic sinusoid endotheliocytes accounts for more than 80% of all liver cells and they perform the majority of liver functions 26. Previous studies have proved that co-culture is better than monoculture for achieving cell-cell interactions, more physiological microenvironments and enhanced hepatocyte functions. Thus, we co-cultured WRL68 cells and LX2 cells on the liver lobule chips. WRL68 cells and LX2 cells cultured in Dulbecco's modified Eagle's medium (DMEM; Gibco Life Technologies, USA) supplemented with 10% fetal bovine serum and 1% penicillin-streptomycin at 37 °C and 5% CO_2_ in an incubator.

### 2.12 Detection of serum biochemical indexes and liver tissue morphology staining

Liver tissue morphology was assessed using photographs of individual mouse livers; liver samples were then fixed in 4% paraformaldehyde for more than 24 h and sent to Wuhan seville biotechnology company for H&E staining and preparation of immunohistochemistry (IHC) sections. The sections were then sealed and observed under a microscope.

### 2.13 Liver BAs profiling using LC-MRM-MS

Thirty-three BAs were weighed and used to prepare a 25 μg/ml mixed standard sample; this standard was diluted with methanol to obtain a series of working mixtures with concentrations of 25,000, 15,000, 5,000, 2,500, 500, 250, 50, 25, 15, 5, 2.5, and 1.5 ng/mL, respectively. A solution of CA-d4, GCA-d4, GCDCA-d4, LCA-d4, and UDCA-d4 were each prepared for use as an internal standard. All BA solutions were stored at -20 °C. Metabolites were extracted after grinding with liquid nitrogen. Samples of equal amounts were weighed and mixed by vortexing. To 100 μL of the diluted sample, 300 μL precipitant (acetonitrile: methanol = 8:2 (v/v) and the mixed internal standard solution were added, and the samples were vortexed, stored on ice for 30 min, and centrifuged at 12,000 rpm for 10 min at 4 °C. The supernatant was collected for LC-MS analysis. An ultra-high performance liquid chromatography coupled to tandem mass spectrometry (UHPLC-MS/MS) system (ExionLC™ AD UHPLC-QTRAP 6500+, AB SCIEX Corp., Boston, MA, USA) was used to quantitate BAs in Novogene Co., Ltd. (Beijing, China). Separation was performed on a Waters ACQUITY UPLC BEH C18 column (2.1×100 mm, 1.7 μm) which was maintained at 50°C. The mobile phase, consisting of 0.1% formic acid and 5mM Ammonium acetate in water (solvent A) and acetonitrile (solvent B), was delivered at a flow rate of 0.35 mL/min. The solvent gradient was set as follows: initial 5% B, 0.5 min; 5-30% B, 1.5 min; 30-37% B, 4 min; 37-38% B, 5 min; 38-39% B, 5.5 min; 39-42% B, 6 min; 42-43% B, 6.5min; 43-50% B, 9.5 min; 50-60% B, 11 min; 60-95% B, 12 min; 95-5% B, 13.1 min; 5% B, 15 min.

The mass spectrometer was operated in negative multiple reaction mode (MRM) mode. Parameters were as follows: IonSpray Voltage (-4500 V), Curtain Gas (30 psi), Ion Source Temp (550 °C), Ion Source Gas of 1 and 2 (60 psi).

### 2.14 Biotin labeling and target fishing

The conjugated target drug-probe molecule (Bio-Lignan) was obtained by synthesizing *Schisandra chinensis* lignans with biotin tags. The important intermediates and resulting products were characterized using nuclear magnetic resonance spectrometry. Next, the probe was incubated with ANIT-induced WRL68 cells and liver tissue lysate for 2 h. Streptavidin beads were used to pull down the proteins bound to the probe. The protein bands were visualized by silver staining, and the target bands were separated and analyzed by protein LC-MS/MS.

### 2.15 Luciferase reporter assay

The plasmids pCDNA3.1(+)-empty, pCDNA3.1(+)-PXR^G144A^, pCDNA3.1(+)-PXR^S106A^, pCDNA3.1(+)-PXR^WT^, and pGL3.1-CMVR were transfected into WRL68 cells for 36 h. Next, the cells were treated with fresh DMEM containing DMSO, RIF (10 μM, S2543-1g), or Sin B (5, 10, and 20 μM) for 24 h. Relative luciferase activity was measured using a dual-luciferase reporter assay system by following the manufacturer's protocols. To construct of a luciferase reporter gene vector containing TFEB promoter, the full-length TFEB promoter containing wild-type (WT) or mutant (Mut) was respectively cloned into pGL3-basic vectors (Genecreate, Wuhan, China). The reporter vector pGL3-TFEB promoter containing wide or mutant type, pRL-TK (renilla luciferase vector) and with or without pcDNA3.1-PXR were co-transfected into HEK293T cells.

### 2.16 Recombinant plasmid construction, protein expression and purification

The gene fragment encoding the human PXR-LBD domain (106-434aa) obtained from human liver cDNA was inserted into pKMH vectors. The recombinant plasmids were then transformed into DH5α *E. coli* cells. Then, sequenced PXR-LBD recombinant plasmids were transferred into BL21-Codonplus (DE3) competent cells and then transferred into *E. coli* for overexpression. 0.2 mM isopropyl-*β*-d-thiogalactoside was used for the induction at 16 °C for 6 h. Cultures were collected and precipitated with a buffer containing 20 mM Tris-HCl (pH 8.0), 500 mM NaCl, 250 mM imidazole, 5% glycerol, 4 mM β-ME, and 0.1 mM PMSF. Proteins were purified from the samples using the Ni-NTA and size-exclusion chromatography (GE, USA); the expression levels and purities of target proteins were evaluated using SDS-PAGE electrophoresis. The proteins were stored at -80 °C.

### 2.17 Crystallization and structural characterization

The PXR-LBD domain (5.0 mg/ml) crystallization conditions were obtained from the Protein Data Bank (PDB). Sample preparation involved containing 50 mM imidazole, 6.5-9.0% isopropyl alcohol, 7.5-10% ethylene glycol, and 0.1 M Tris-HCl pH7.5. Crystallization was carried out using a vapor diffusion approach. Crystals were grown at 4 °C for two days, then quickly frozen in liquid nitrogen for X-ray diffraction analysis, which was performed by a XtaLAB Synergy system. Several refinement cycles were performed, and Coot was used to model the final protein structure **(Table 2)**.

### 2.18 Isothermal titration calorimetry and Surface Plasmon Resonance (SPR) analysis

ITC analysis used PXR-LBD proteins exchanged into PBS and Sin B was dissolved in DMSO. The titration was conducted on an auto-ITC instrument at 25 °C. 5 mM Sin B was aspirated into the composite needle and titrated into the sample cells containing 140 μM PXR-LBD by 19 injections. MicroCal Origin was used to analyze and process the data. A SPR assay was performed on a Biacore 8K system (Biacore, GE Healthcare, Boston, MA, USA). The PXR-LBD proteins were fixed on a CM5 chip, and the optimal pH for enrichment was maintained at pH 5. Sin B was dissolved in PBS containing 1% DMSO, and real-time molecular interactions were quantified using a drug concentration gradient. GraphPad was used to plot the results.

### 2.19 Cellular thermal shift (CETSA) and Drug affinity responsive target stability (DARTS) assay

CETSA assays were used WRL68 cells, and they're treated with RIPA and incubated with the proteins and 10 μM Sin B. The control sample contained DMSO solution of the same volume; samples were evenly mixed and incubated at room temperature for 1.5 h. A PCR machine was used to create the thermal gradients, which were 40-56 °C for the Sin B-PXR sample. The samples were then centrifuged at 12,000 rpm for 15 min at 4 °C. 20 μL of supernatant was combined with 5 × loading buffer to prepare samples for western blotting. CETSA assays were treated with T-PER containing 1% PMSF and 1% PPI in WRL68 cells. The extracted proteins were divided into a control sample and a 10 μM Sin B sample. The control sample contained DMSO of an equal volume, which was evenly mixed and incubated at room temperature for 1 h. Next, pronase was dissolved with 1x TNC to a final concentration of 1.25 μg/μL. The ratios of the enzyme-to-total protein in the Sin B-PXR assays were adjusted to 1:200, 1:400, or 1:800, respectively. All samples were homogenized, incubated at room temperature for 15 min. followed by western blotting after the addition of the 5 × loading buffer.

### 2.20 Evaluation of the PXR stability

The protein amplitude size, mass distribution and static light scattering indexes of the PXR protein treated with Sin B were evaluated by an Uncle protein stability analyzer. PXR protein (0.5 mg/ml) was incubated with 5, 10 and 20 μM Sin B on ice for 30 min, respectively, along with a DMSO control group. A high-speed centrifugation at 12,000 rpm for 2 min was performed after the incubation before the T_m_ values were measured.

### 2.21 Molecular docking and molecular dynamics simulations

The structure of the PXR-LBD was obtained from the PDB database (PDB ID: 1NRL), and the 3D structure of Sin B was obtained from the NCBI database (PubChem CID: 158103). AutoDock was used to predict the binding mode of Sin B to PXR-LBD, and PyMOL was used to visually analyze the docked complex. Next, the crystal structure of PXR was selected as the initial structure for building a computational model. Schrödinger's Maestro portal (version 11.1) (Schrödinger, LLC, New York, NY, 2017) was used to determine the protonated state of the protein and prepare the ligand. Residues near the ligand were checked manually. The Glide module was utilized to perform molecular docking and to select poses for subsequent MD. The protein and ligand were separately parameterized with Amber ff14SB 27 and general AMBER force field (GAFF) 28. The restrained electrostatic potential (RESP) charge of the ligand was calculated at the HF/6-31G (d) level. All complex systems were solved into cuboid TIP3P water boxes with an extended distance to the protein boundary of 10 Å. Sodium ions were added to maintain charge neutrality.

Initially, three minimization steps were performed on each system. First, only the solvent molecules were minimized while applying a 100 kcal^-1^∙mol∙Å^-2^ restraint force to the protein and ligand. In the second step, residue and solvent sidechains were minimized by applying a 100 kcal^-1^∙mol∙Å^-2^ force to the backbone atoms of the protein and ligand. In the third step, all atoms were minimized without any restraints. These three minimization steps consisted of 4,000 steps using the steepest descent algorithm and 2,000 steps using the conjugate gradient algorithm (for 6,000 steps total). Next, systems were heated from 0 K to 300 K over 500 ps, with the time step set to 1 fs under an NVT ensemble. The temperature increased from 0 K to 300 K within the first 400 ps, and a temperature of 300 K was maintained from 400 ps to 500 ps via a 25 kcal^-1^∙mol∙Å^-2^ force applied to all atoms. After heating, 500 ps of density equilibration simulations were performed with a restraint of 25 kcal^-1^∙mol∙Å^-2^, followed by another 500 ps of unrestrained density equilibration simulations (1 fs time-step at 300 K and 1.0 atm pressure). Finally, 500-ns production simulations were carried out for all systems under the NVT ensemble, with a 2-fs time step and the entire system relaxed. The temperature was controlled via Langevin dynamics. The cutoff for nonbounded interactions was set to 10 Å. The particle-mesh Ewald method was applied to long-range electrostatic interactions. Hydrogen atoms were constrained using the SHAKE algorithm. All MD simulations were performed with the PMEMD program belonging to the Amber20 package 29. Trajectory analysis was performed using the CPPTRAJ module.

### 2.22 Statistical analyses

For western blot and IF images obtained in this study, Image J was used to create grayscale images and to determine fluorescence intensity; GraphPad Prism 9.0 (GraphPad Software, San Diego, CA, US) was used for plotting. All experimental data were expressed as means ± SEMs or means ± SDs of three independent experiments. Statistical comparisons of two groups were performed using unpaired *t*-tests. One-way ANOVAs were used to assess differences among groups. *P*-values < 0.05 were considered statistically significant.

## 3. Results

### 3.1 The abnormal accumulation of BAs aggravates hepatocyte injury

**Figure 1:**
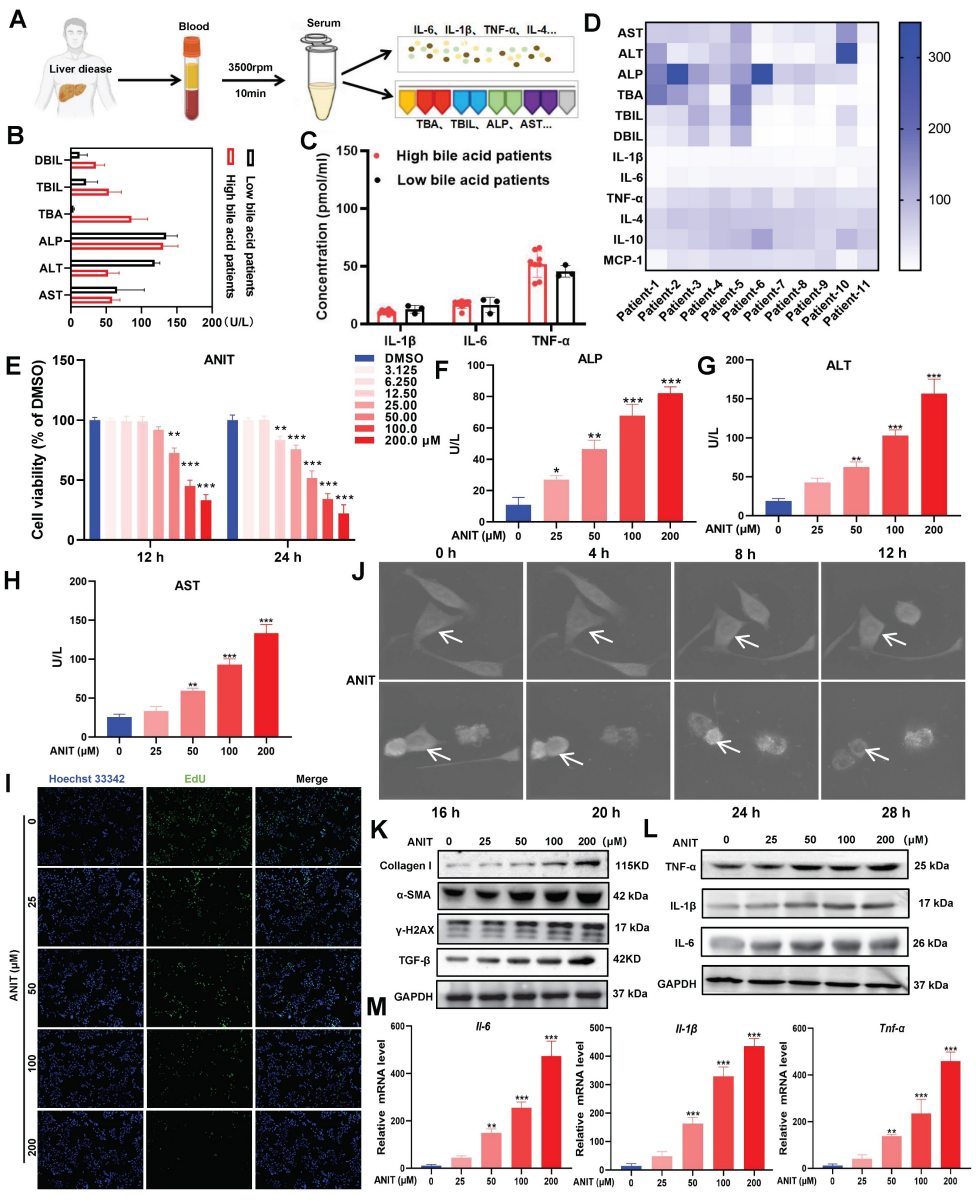
** BAs accumulation promotes hepatocyte injury.** (A) Clinical sample collection flow chart. (B) Serum DBIL, TBIL, TBA, ALP, ALT, and AST levels in clinical samples. (C) Concentration of inflammatory factors in clinical samples as detected by ELISA. (D) Heat map of liver injury with high BA versus low BA liver disease in clinical samples. (E) MTT assay of ANIT-treated WRL68 cells. (F-H) Levels of ALP, ALT, and AST in WRL68 cells after treatment with ANIT. (I) Effects of ANIT treatment on WRL68 cell self-repair and proliferation as determined via EdU assay (20x; scale=25 μm). (J) NanoLive 3D live cell imaging was used to analyze the effects of 50 μM ANIT on WRL68 cell morphology at different time periods. (K) Western blot to detected the α-SMA, Collagen I, TGF-β and γH2AX expression levels in WRL68 cells. (L) Western blot of TNF-α, IL-1β, and IL-6 after ANIT treatment. (M) RT-qPCR quantification of the inflammatory cytokines *Il-6*, *Il-1β*, and *Tnf-α* in WRL68 cells after ANIT treatment. At least three replicates were used for each trial. Statistical comparisons were made between each ANIT treatment and the control, ^*^P < 0.05, ^**^P < 0.01, ^***^P <0.001.

Patients with clinical liver disease end up having elevated BA and bilirubin levels. In this study, we quantified the indicators of liver damage and inflammation in 11 clinical liver disease samples **(Figure 1A)**. Heatmap analysis revealed that direct bilirubin (DBIL), total bilirubin (TBIL), total bile acids (TBA), ALP, ALT, and AST were more abundant in blood samples from patients with high BA levels** (Figure 1B)**. Moreover, the levels of the pro-inflammatory cytokines interleukin (IL)-1β, IL-6, and tumor necrosis factor (TNF)-α were higher in patients with high BA levels than those in patients with low BA levels** (Figure 1C-D)**. These results suggest that patients with liver disease tend to have BA accumulation, leading to inflammation. To determine the adverse effects of BA accumulation, we treated WRL68 cells with α-naphthyl isothiocyanate (ANIT) at doses between 0-200 μM for 12 h and 24 h. The MTT assay showed that treatment with 50 μM ANIT for 12 h significantly decreased the survival rate of WRL68 cells to 40%, with further decreases in survival observed in a dose- and time-dependent manner (**Figure 1E**). In addition, ANIT treatment increased the levels of ALP, ALT, and AST in a dose-dependent manner **(Figure 1F-H)**. EdU assays showed that 50 μM ANIT significantly inhibited hepatocyte proliferation **(Figure 1I)**. We used NanoLive 3D cell imaging to analyze the effects of 50 μM ANIT on the morphology of WRL68 cells at different time points. We observed significant changes in WRL68 cell morphology after 8 h of ANIT treatment, with the cells gradually becoming spherical and dying after 12 h **(Figure 1J, Figure S1)**. Next, we investigated the effects of ANIT on DNA damage and fibrosis. Western blotting revealed that ANIT treatment substantially promoted the expression of γ-H2AX, α-SMA, Collagen I, and TGF-β1 in a dose-dependent manner (**Figure 1K**), supporting the hypothesis that BA accumulation promotes DNA damage and fibrosis in hepatocytes. Finally, we evaluated the effect of ANIT on the expression of inflammatory cytokines and found that both the mRNA and protein levels of intracellular IL-6, IL-1β, and TNF-α increased in a dose-dependent manner **(Figure 1L-M)**. Taking together, these results indicate that the abnormal accumulation of BAs can aggravate hepatocyte injury and promote inflammation.

### 3.2 Sin B protects hepatocytes from ANIT-induced damage

**Figure 2:**
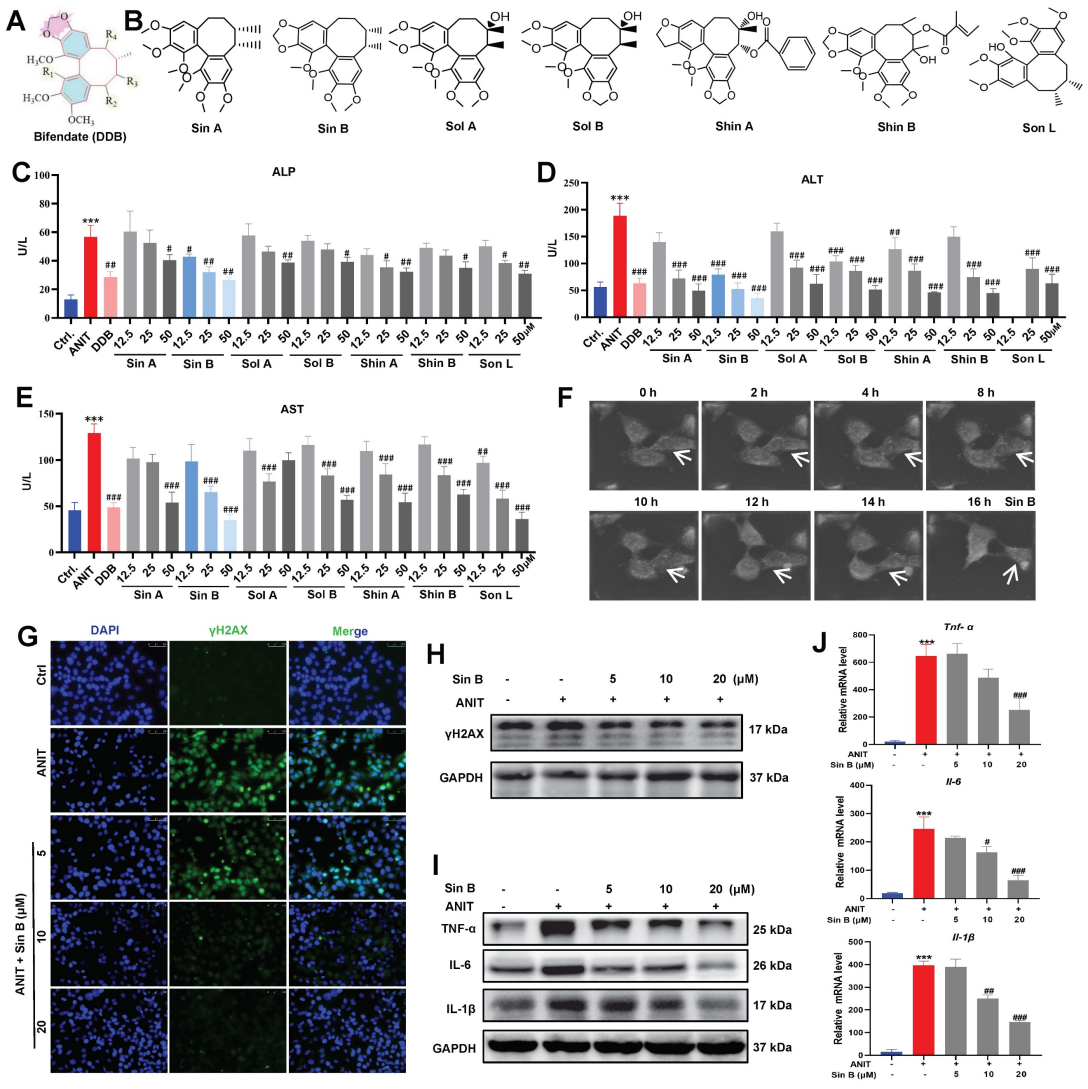
** Sin B protects hepatocytes from ANIT-induced damage.** (A) Bifendate (DDB) structure. (B) Seven compounds identified in *Schisandra chinensis* extracts with the same basis structure as DDB, namely Sin A, Sin B, Sol A, Sol B, Shin A, Shin B, and Son L. (C-E) Assay screening the effects of the seven lignans on ALP, ALT, and AST levels. (F) NanoLive 3D live cell imaging for analyzing the effects of Sin B on WRL68 cell morphology at different time periods. (G-H) Immunofluorescence and western blot for the γH2AX protein level assesment after the Sin B treatment. (I-J) Expression of IL-6, IL-1β and TNF-α as analyzed by western blot and RT-qPCR. All experiments were repeated three times, and *t*-tests were used to determine statistical significance for comparisons between the control group and ANIT groups (^**^P < 0.01, ^***^P <0.001); compaired with the ANIT group and Sin B groups (^#^*P* < 0.05, ^##^*P* < 0.01, ^###^*P* <0.001).

Bifendate (DDB), a drug used to treat viral hepatitis and drug-induced liver injury, significantly reduces serum ALT elevation caused by various chemical poisons and enzymes **(Figure 2A)**. We screened seven lignans from Schisandra chinensis extract according to their similarity to DDB structure, including Sin A, Sin B, Shin A, Shin B, Sol A, Sol B, and Son L **(Figure 2B)**. To assess the protective effect of lignans on CLI, we first used 50 μM ANIT to treat WRL68 cells for 12 h to establish a CLI model. As depicted in **Figure 2C-E**, all seven lignans inhibited the elevations in ALP, ALT, and AST to varying degrees. Specifically, 12.5 μM Sin B significantly reduced ALP levels, while none of the other compounds showed significant differences at the same dosage. Moreover, 12.5 μM Sin B significantly reduced ALT levels. Aside from Shin A, which also showed a significant difference at 12.5 μM, no other compounds exhibited significant effects. Furthermore, 25 μM Sin B and 12.5 µM Son L significantly reduced AST levels. Thus, the overall efficacy of Sin B was more pronounced than that of the other components. NanoLive 3D live analysis revealed that Sin B maintained cell morphology for 16h **(Figure 2F)**. Moreover, western blotting and immunodeficiency assays demonstrated that 20 μM Sin B significantly reduced γH2AX expression **(Figure 2G-H)**. Finally, we investigated the effects of Sin B on hepatocyte inflammation and found that Sin B significantly reduced both the mRNA and protein levels of IL-6, IL-1β, and TNF-α **(Figure 2I-J)**. In summary, Sin B mitigated ANIT-induced damage by alleviating inflammation and maintaining cell morphology.

### 3.3 Sin B administration significantly attenuates CLI injury

**Figure 3:**
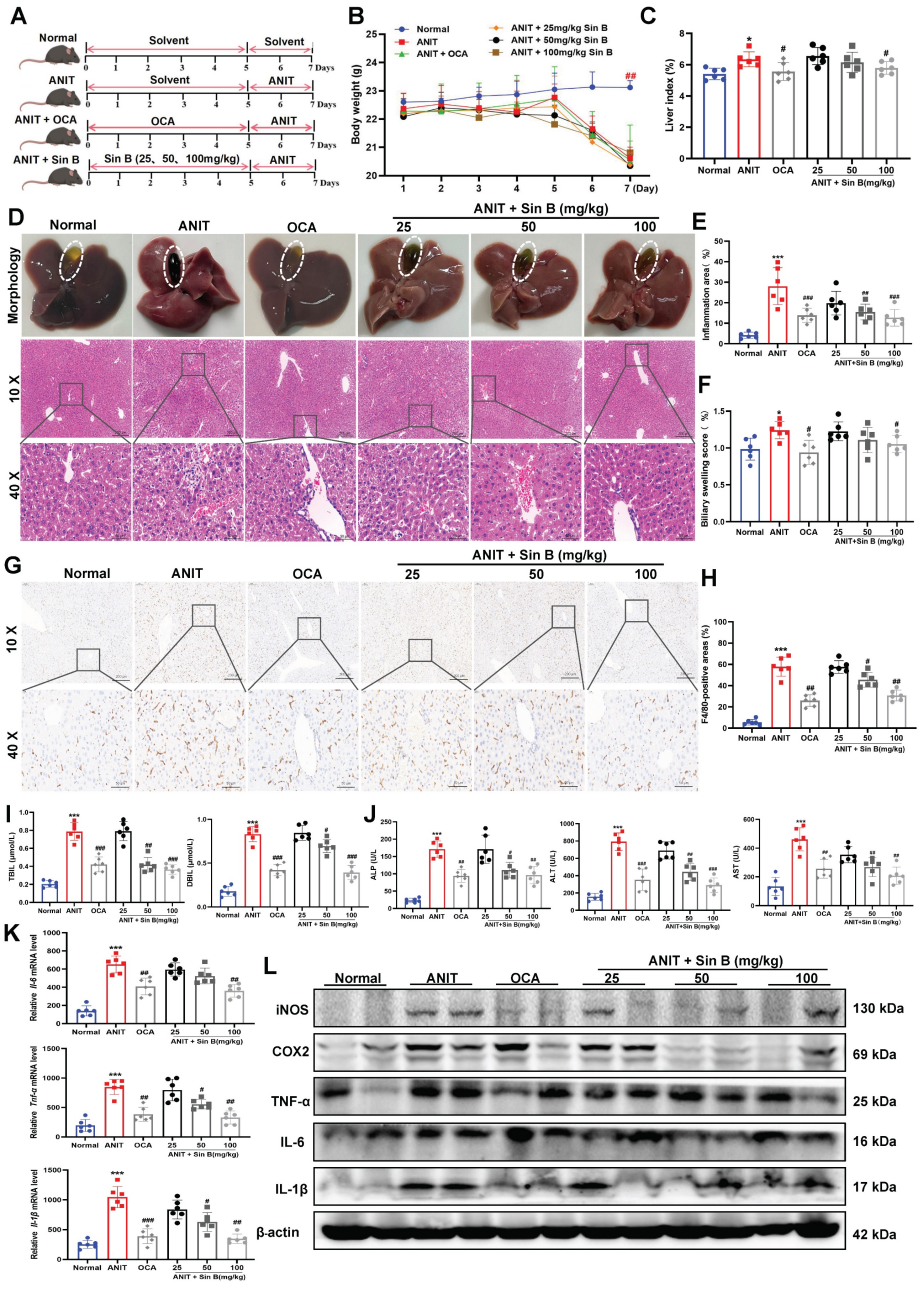
** Sin B alleviates CLI inflammation *in vivo.*** (A) Detailed timeline of CLI model induction via ANIT and the use of Sin B. (B-C) Body weight and liver index for each group, with at least six animals per group. (D) Morphology and H&E staining of liver tissues from different groups of mice (10x, scale=200 μm; 40x, scale=50 μm). The gallbladder is circled by an elliptical dotted line. (E-F) The proportion of inflammatory area and the degree of bile duct swelling as measured by H&E staining. (G-H) IHC stain of F4/80 expression in liver tissues (10x, scale=100 μm; 40x, scale=20 μm). (I-J) Serum TBIL, DBIL, ALP, ALT, and AST levels as quantified for each group. (K) Expression of the inflammatory factors *Il-6*, *Tnf-α, and Il-1β* in liver as quantified via RT-qPCR. (L) Western blot analysis of the iNOS, COX2, TNF-α, IL-6, and IL-1β levels after Sin B treatment. All experiments were repeated six times, and *t*-tests were used to determine statistical significance in comparisons of the ANIT group and nornal group (^*^P < 0.05, ^***^P <0.001) and of the ANIT group versus Sin B group (^#^*P* < 0.05, ^##^*P* < 0.01, ^###^*P* <0.001).

To verify these effects *in vivo*, we established a C57BL/6J mouse model of CLI **(Figure 3A)**. The weight of mice in the ANIT-treated group was significantly decreased, whereas that of mice treated with Sin B (100 mg/kg) slightly increased **(Figure 3B)**. Compared to the ANIT group, the liver index was significantly decreased in mice administered with 100 mg/kg Sin B **(Figure 3C)**. Morphological analyses of the liver revealed swollen liver tissues and more hemorrhagic points after ANIT treatment, and the gallbladder was dark brown due to BA accumulation. After treatment with OCA, the number of hemorrhagic points significantly decreased, and the color of the gallbladder returned to normal. After Sin B treatment, liver tissue surface adhesions and the number of hemorrhagic points decreased in the 50 and 100 mg/kg groups, while gallbladder color tended to return to normal, and BA accumulation was significantly reduced. Hematoxylin and eosin (H&E) staining revealed that ANIT treatment increased inflammatory infiltration and cell fibrosis, accompanied by bile duct cell destruction. However, these effects were alleviated by treatment with 50 and 100 mg/kg Sin B, resulting in bile duct swelling and decreased inflammatory cell infiltration **(Figure 3D-F)**. IHC evaluation revealed higher expression of F4/80 in the ANIT-treated group than that in the control group. However, 50 and 100 mg/kg Sin B significantly reduced the elevated F4/80 levels** (Figure 3G-H)**. Additionally, serum TBIL and DBIL levels were reduced by Sin B treatment in a dose-dependent manner **(Figure 3I)**. Similarly, ALP, ALT, and AST levels were reduced by Sin B treatment **(Figure 3J)**, and the secretion of IL-6, IL-1β, and TNF-α was also reduced in a dose-dependent manner **(Figure 3K)**. Further, the protein levels of COX2, IL-1β, IL-6, iNOS, and TNF-α were significantly higher in the ANIT group, with Sin B treatment blocking the elevation of these protein levels **(Figure 3L)**. In summary, Sin B reduced ANIT-induced cellular injury* in vivo* by inhibiting inflammation.

### 3.4 Sin B upregulates the expression of metabolic enzymes to promote BA metabolism

**Figure 4:**
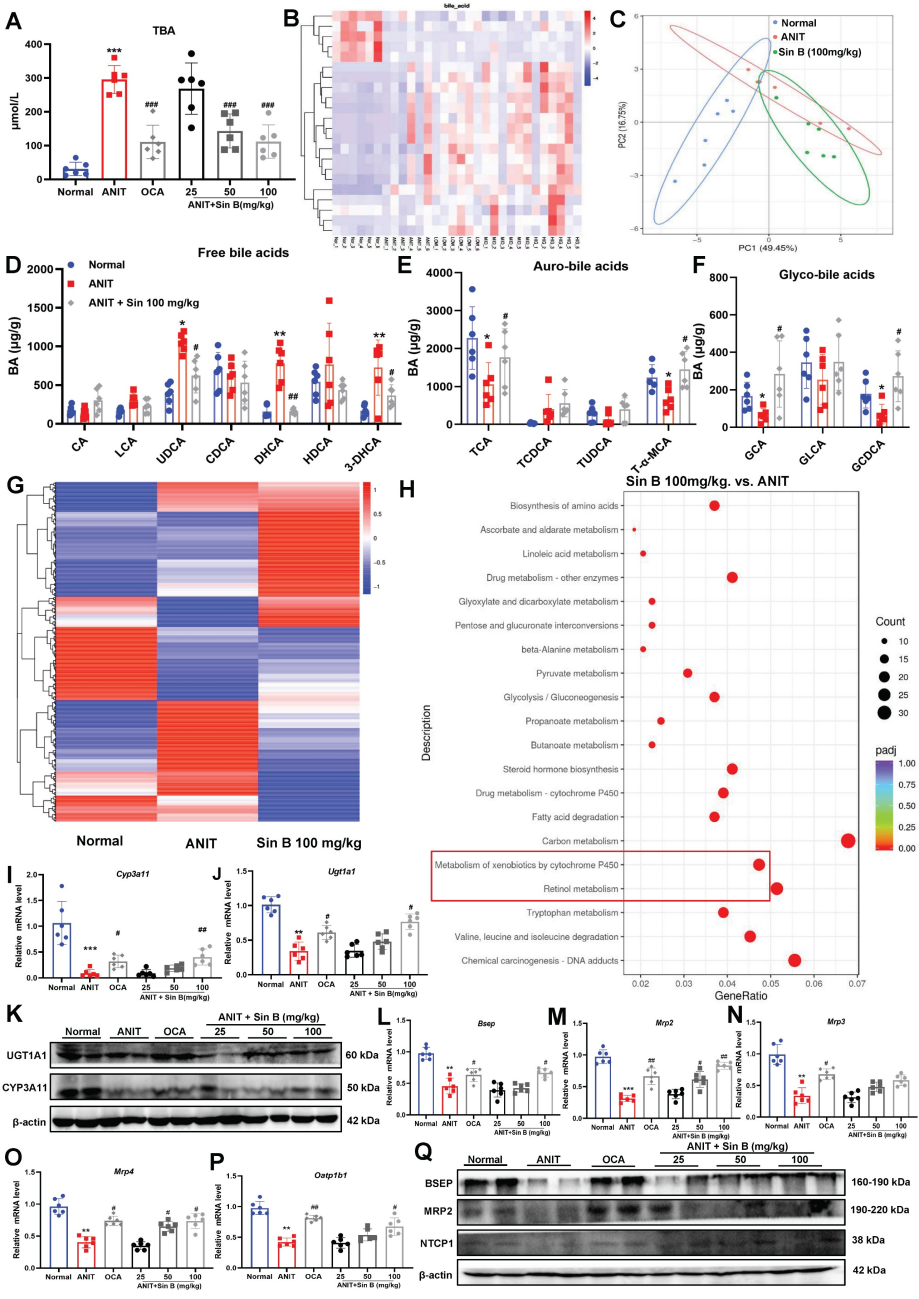
** Sin B regulates BA metabolism through the cytochrome P450 pathway.** (A) TBA levels of each group. (B-C) LC-MRM-MS determination of BAs types and abundance in normal, ANIT, and Sin B 100 mg/kg groups; these data were used to create a heat map and PCA plot. (D-F) Free BAs, tauro-BAs, and glyco-BAs level in normal, ANIT, and Sin B 100 mg/kg groups. (G) Heat map of differentially expressed genes in the normal, ANIT, and Sin B 100 mg/kg groups. (H) KEGG pathway enrichment analysis for differentially expressed genes in the ANIT versus Sin B 100 mg/kg group. (I-K) mRNA and Protein expression level of the metabolic enzymes CYP3A11 and UGT1A1 in liver, as assessed via western blotting and RT-qPCR, respectively. (L-P) Expression of the BA metabolism and transport genes *Bsep*, *Mrp2*, *Mrp3*, *Mrp4*, and *Oatp1b1* was assessed by RT-qPCR. (Q) Expression of the BA metabolism and transport proteins BSEP, MRP2, and NTCP1 was assessed by western blotting. All experiments were repeated six times, and *t*-tests were used to determine statistical significance in comparisons of the ANIT group and normal group (^*^P < 0.05, ^**^P < 0.01, ^***^P < 0.001) and ANIT versus Sin B groups (^#^*P* < 0.05, ^##^*P* < 0.01, ^###^*P* < 0.001).

CLI is aggravated by abnormal BA metabolism, excretion, and retention *in vivo*. Measurement of TBA levels in liver tissues revealed that BA levels were 5.6-fold higher in the ANIT group but were reduced by 50 mg/kg Sin B **(Figure 4A)**. LC-MRM-MS metabolic assays further demonstrated that the types and abundance of BAs differed between the control, ANIT, and Sin B groups **(Figure 4B-C)**. After ANIT treatment, the abundance of free BAs significantly increased, including 3-dehydrocholic acid (3-DHCA), dihydrochalcone acid (DHCA), and ursodeoxycholic acid (UDCA). In contrast, Sin B increased the levels of partially bound BA, including taurine-amidated α-MCA (T-α-MCA), glycocholic acid (GCA), glycochedeoxycholic acid (GCDCA), and taurocholic acid (TCA) **(Figure 4D-F).** The shift towards conjugated BAs (like T-α-MCA and GCA) is a recognized detoxification mechanism. The conjugation increases water solubility, reduces cytotoxicity, and facilitates biliary excretion. To further explore how Sin B mediates BA homeostasis, we performed transcriptomic sequencing for control, ANIT-, and Sin B- (100 mg/kg)-treated groups. As shown in **Figure 4G**, gene expression levels were significantly different among the three groups. Pathway enrichment analysis of the differentially expressed genes using the kyoto encyclopedia of genes and genomes (KEGG) database revealed that Sin B was mainly involved in cytochrome P450-related metabolic pathways, drug metabolism, and toxic substance metabolism, with the strongest correlation in the P450 metabolic pathways **(Figure 4H)**. RT-qPCR revealed that the levels of *CYP3A11* and *Ugt1a1*, which are involved in BA metabolism via the P450 pathway, were significantly decreased in the ANIT group, whereas they were increased in the other two groups, with the most significant effect observed at a Sin B dose of 100 mg/kg **(Figure 4I-K)**. Further, we evaluated the expression levels of the BA-metabolizing enzymes and transporter proteins bile salt export pump (*Bsep*), *Mrp2*, *Mrp3*, *Mrp4*, and organic anion-transporting polypeptide 1B1 (*Oatp1b1*) in liver tissues using RT-qPCR to investigate how Sin B regulates BA metabolism. The results indicated that Sin B increased the expression of these transporter genes, with 100 mg/kg Sin B showing a stronger regulatory effect on *Mrp2* expression than OCA** (Figure 4L-P)**. Additionally, the expression levels of BSEP, MRP2, and Na^+^/taurocholate co-transporter 1 (NTCP1), which were reduced by ANIT treatment, were restored by Sin B treatment **(Figure 4Q)**. verall, these findings demonstrate that Sin B promotes BA metabolism by upregulating the expression of P450 metabolic enzymes and transporter proteins, ultimately reducing hepatocyte damage *in vivo*.

### 3.5 Sin B promotes BAs metabolism and improves CLI in liver lobule chips

**Figure 5:**
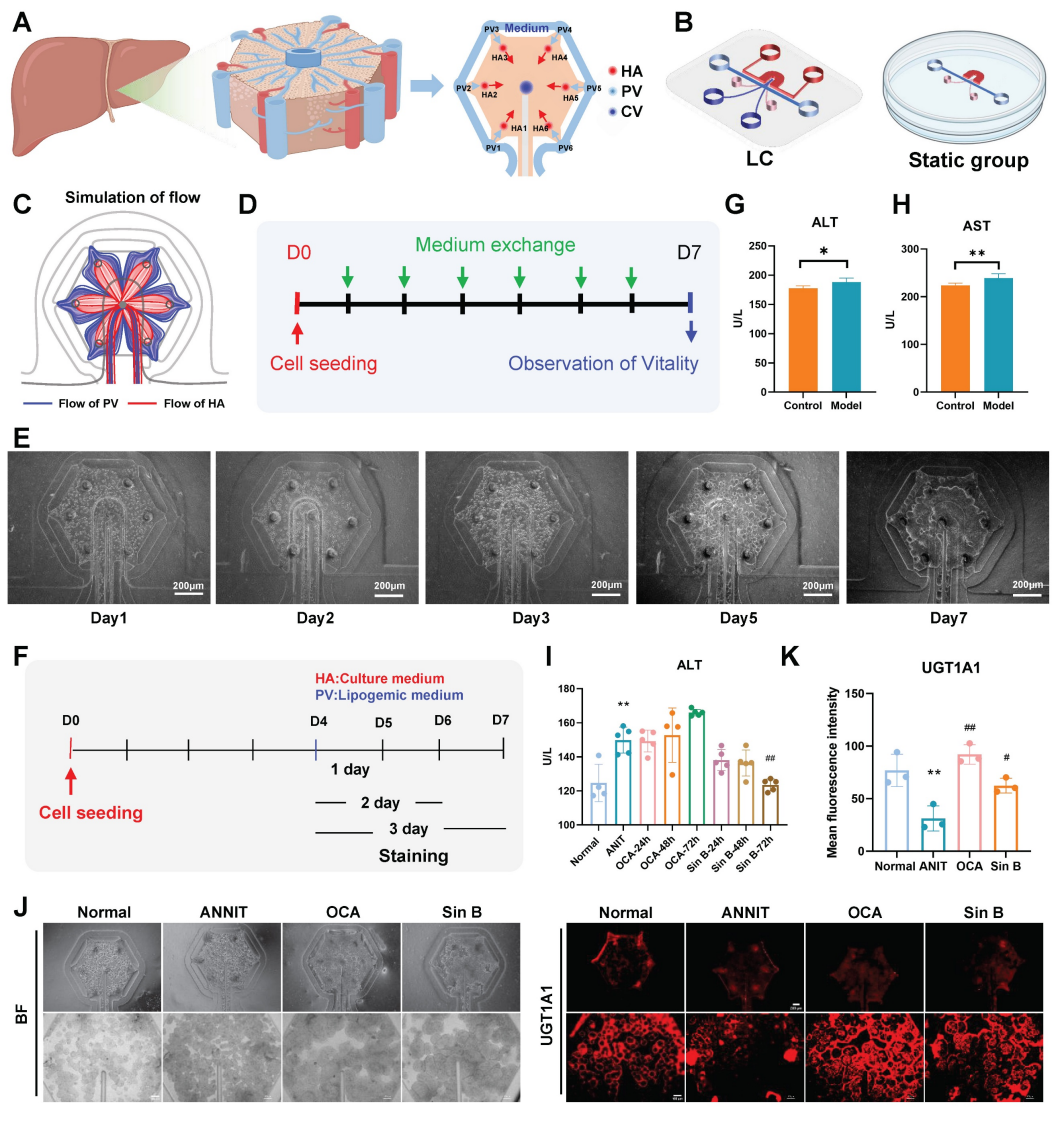
** Sin B improves CLI on liver lobule chips.** (A)schematic illustration of a single liver lobule and its simplified structure. (B) the expanded view of the liver lobule chips. (C) the simulated flow in the liver lobule chips. (D) the protocol for cell culture and analysis. (E) the images of liver lobule chips. (F) the protocol for inducing CLI. (G-H) The level of ALT and AST in the liver lobule chips. (I) ALT levels at different time periods. (J-K) Immunofluorescence for the UGT1A1 protein level assesment after the Sin B treatment. All experiments were repeated six times, and *t*-tests were used to determine statistical significance in comparisons of the ANIT group and normal group (^*^P < 0.05, ^**^P < 0.01, ^***^P < 0.001) and ANIT versus Sin B groups (^#^*P* < 0.05, ^##^*P* < 0.01, ^###^*P* < 0.001).

We designed the liver lobule chips according to the functional unit of the liver, liver lobule, and hexagonal tissue with a CV at the center, and HA and hepatic PV at each of the six corners **(Figure 5A)**. The designed liver lobule chips consisted of three PDMS layers **(Figure 5B)**: the top layer with the flow pathways of the HA (red) and the CV (deep blue), the middle layer (thickness; 50 μm) with six HA micro-holes (diameter; 100 μm), one CV micro-hole (diameter; 100 μm), two PV holes (diameter; 3 mm) and two holes for loading cell-laden extracellular matrix (diameter; 1.5 mm) and the bottom layer with a hexagonal cell culture zone (pink) and a flow pathway of the PV (light blue). The simulation results showed that the medium flowing through the PV and HA flowed radially through the cell culture zone **(Figure 5C)**. The cells showed high viability for at least seven days in the liver lobule chips **(Figure 5D-E)**. We constructed a liver CLI chip model to verify the effects of Sin B *in vitro*** (Figure 5F)**. ANIT treatment (100 μM) for 24 hours significantly increased the levels of ALT and AST in the liver lobule chips, indicating the successful construction ofa liver lobule chip model for CLI **(Figure 5G-H)**. Compared to the ANIT group, ALT levels were significantly decreased in liver lobule chips with 20 μM Sin B treatment for 72 h **(Figure 5I)**. I IF evaluation revealed lower expression of UGT1A1 in the ANIT-treated group than that in the control group. However, Sin B (20 μM) significantly increased UGT1A1 levels **(Figure 5J-K)**.

### 3.6 Sin B directly interacts with PXR-LBD and promotes PXR nuclear translocation

**Figure 6:**
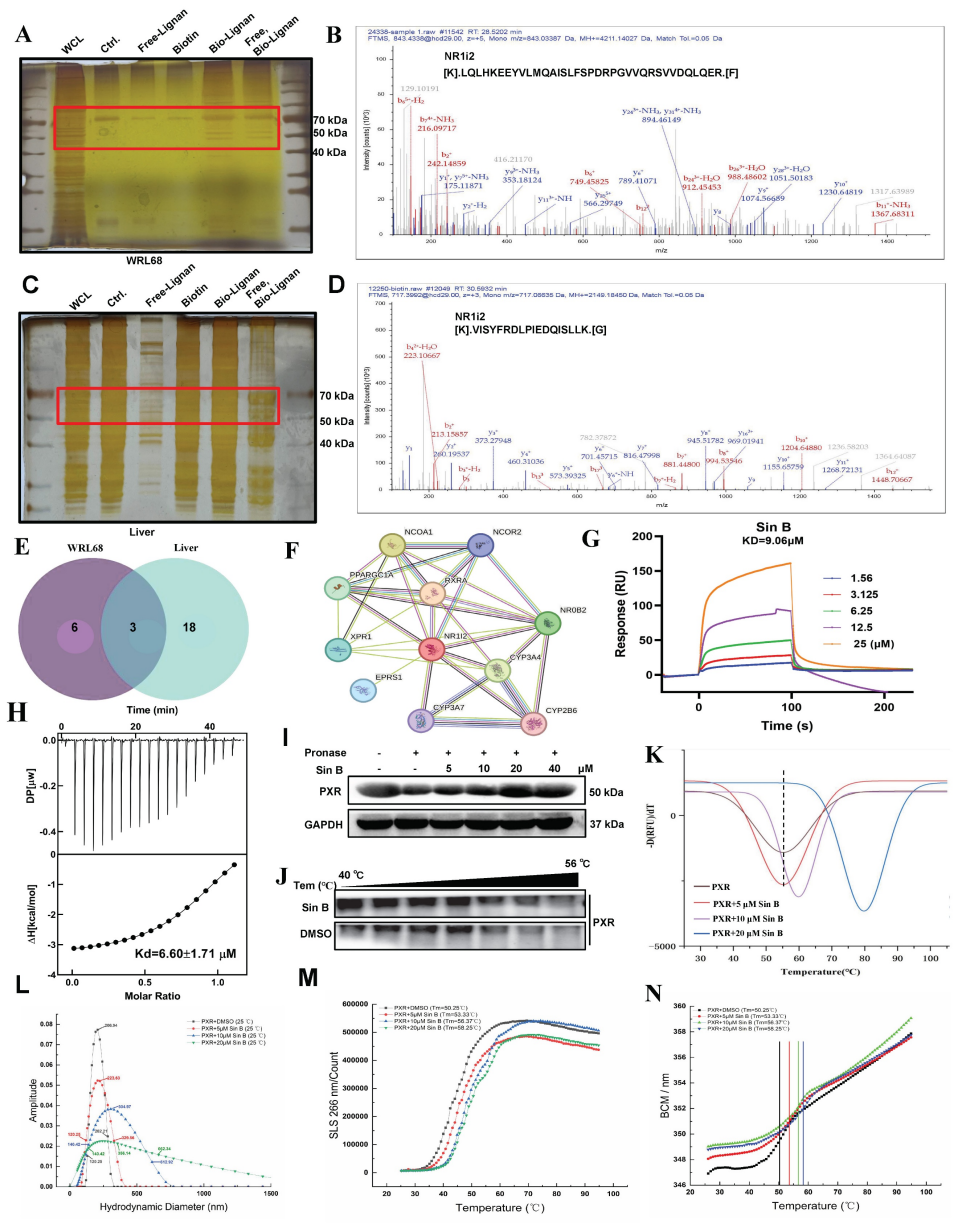
** Sin B directly binds PXR.** (A-B) Target fishing and LC-MS/MS results of WRL68 cells with silver staining. Group name abbreviations are as follows: WCL, cell lysate; Ctrl., combined with beads; Free-lignan, free lignan; Biotin, bio-lignan probe; Free, Bio-Lignan: competitive group of free lignans and pentathlon lignan probes. (C-D) Target fishing and LC-MS/MS results of liver tissues with silver staining. (E) Venn diagram of proteins identified by WRL68 cells and liver tissues, and the intersection of proteins is NR1i2(PXR). (F) Interaction network analysis of intersection targets. (G) SPR analysis of Sin B and PXR binding. (H) The binding affinity of Sin B and PXR was determined by ITC. (I-J) CETSA and DARTS assays to determine Sin B affects PXR stability. (K) The effect of Sin B on the stability of PXR by protein heat transfer. (L) The effect of Sin B on particle size of PXR protein. (M-N) The effect of Sin B on the static light scattering and denaturation curve of PXR protein as analyzed by an Uncle protein stabilizer.

Next, we evaluated the hepatoprotective effects of Sin B in CLI and aimed to identify its therapeutic targets. A biotin-labeled version of Sin B was synthesized for the pull-down assays. Target fishing experiments were performed using ANIT-induced WRL68 cells and liver tissues, followed by silver staining to identify candidate targets **(Figure 6A, C)**. Subsequent LC-MS/MS analysis identified PXR as its major target **(Figure 6B, D)**. Cross-target analysis of the top 16 potential targets in WRL68 cells and the top 18 targets in liver tissue revealed that NR1i2 binds to Sin B **(Figure 6E, Figure S2)**.

To determine the direct targets of Sin B in the CLI models, potential Sin B targets associated with cholestatic liver disease were assessed using the OMIN database combined with network pharmacology. The results showed that NR1i2 (PXR) was located at the center **(Figure 6F)**. TTo further evaluate the binding affinity, we used ITC to detect binding in the presence of Sin B, which revealed that PXR binds to Sin B with a *K*_d_ of 6.6 μM while SPR analysis indicated an extracellular binding affinity of 9.1 μM **(Figure 6G-H)**. The target engagement of drugs in cells can be detected using the CESTA or DARTS assays, which can monitor the binding behavior of Sin B to PXR. DARTS analysis showed that Sin B enhanced the thermostability of PXR **(Figure 6I)**, whereas western blot analysis of CESTA indicated increased stabilization of PXR during proteolysis when the WRL68 cell lysate was treated with Sin B** (Figure 6J)**. Furthermore, Sin B enhanced the stability of the PXR protein, as illustrated by protein heat transfer experiments *in vitro* **(Figure 6K)**. Meanwhile, mass distribution of PXR-LBD using the Uncle protein stabilizer showed that the particle size of PXR-LBD was 206.54 nm, and it gradually increased to 356.14 nm after 20 μM Sin B treatment **(Figure 6L)**. The aggregation and denaturation curves showed similar findings **(Figure 6M-N)**. Taken together, these results indicate that Sin B binds directly to PXR and enhances its stability.

### 3.7 Sin B induces PXR conformational changes by binding to S106 and G144

**Figure 7:**
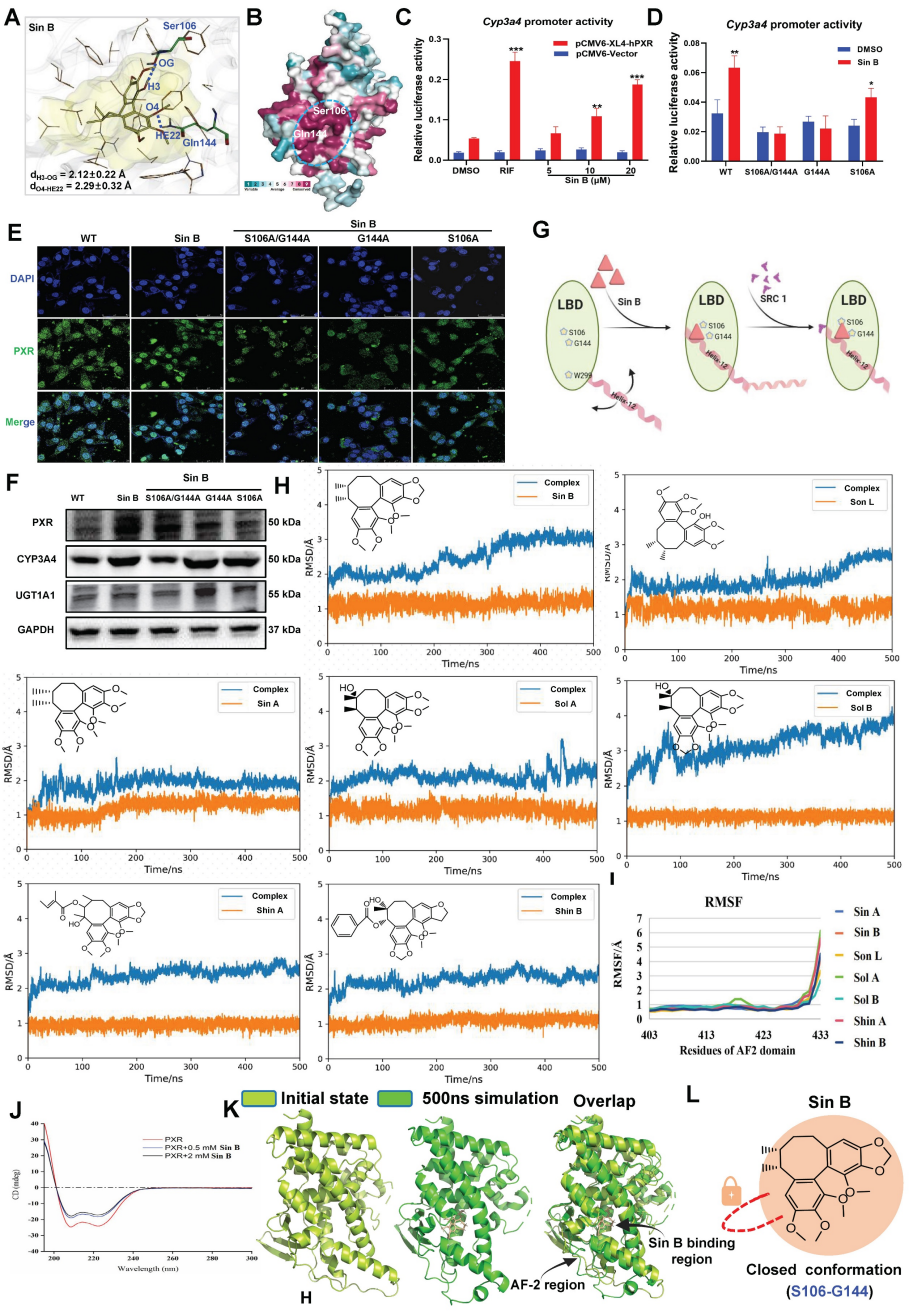
** Targeting of PXR by Sin B induces conformational changes.** (A) Molecular docking analysis of key interaction sites for Sin B and the PXR protein. (B) Sequence conservation analisis of PXR (the conservations scales were shown in lower right). (C) *Cyp3a4* promoter activity as determined by dual-luciferase reporter assays. (D) The effects of Sin B on *Cyp3a4* promoter activity for both unaltered PXR (wild-type) and PXR with key site mutations (S106A/G144A, G144A or S106A) as detected by dual luciferase reporter assays. (E) The effect of Sin B on PXR nucleation after mutation. (F) Western blot detection of CYP3A4 and UGT1A1 expression after mutation of PXR key sites. (G) Schematic diagram of conformational changes in PXR induced by Sin B binding to key sites in the PXR-LBD domain. (H-I) RMSD and RMSF of PXR- lignans analyzed via molecular dynamics simulations. (J) Sin B effects PXR protein conformation as analyzed by circular binary chromatography. (K) Overlay structure of PXR initial state (light green) and Sin B binding PXR 500ns simulation (green). (L) Diagram of the Sin B-PXR complex, illustrating the closure of the binding pocket. All experiments were repeated 3 times, and *t*-tests were used to determine statistical significance when comparing Sin B to DMSO treatments (^**^P < 0.01, ^#^*P* < 0.05, ^##^*P* < 0.01).

We used molecular docking to identify the direct binding sites of Sin B on PXR and found that Sin B interacted with the side chains of Gln144 and Ser106, with interaction distances of 2.1 Å and 2.3 Å, respectively** (Figure 7A)**. Further analysis of the conserved amino acid sequence of the PXR protein showed that these two interaction sites belong to the PXR LBD and are highly conserved **(Figure 7B)**. As a downstream target gene of PXR, the activity of the *Cyp3a4* promoter was investigated using a dual-luciferase reporter assay. As shown in **Figure 7C**, Sin B significantly increased the activity of *Cyp3a4* in a dose-dependent manner. Next, we created key mutants of PXR proteins (S106A/G144A, G144A, and S106A) and transfected them into WRL68 cells. Dual-luciferase reporter assay demonstrated that the transcriptional effects of the *Cyp3a4* promoter were reduced after Sin B treatment **(Figure 7D)**. Additionally, Sin B activation of PXR was significantly decreased by the mutations, as were the downstream protein levels of CYP3A4 and UGT1A1 **(Figure 7E-F)**. A thorough analysis of the binding mode of Sin B revealed that the o-diphenol hydroxyphenyl ring of Sin B may lock the binding pocket of PXR and promote its affinity for Sin B **(Figure 7G)**. In summary, S106 and G144 are key residues involved in the interaction between Sin B and PXR.

To further investigate lignan interactions, MD simulations were performed to determine their binding to PXR-LBD. Root-mean-square deviation (RMSD) analysis revealed relatively stable lignan binding to PXR. Fluctuations in the PXR conformation were also recorded, especially for Sin B and Sol B binding **(Figure 7H, Figure S3)**. These conformational changes were localized to the tail of helix-12 (residues 403-433) upon lignan binding. Trajectory analysis showed that hydrogen bonds were frequently formed between the lignans and PXR-LBD, with a relatively short interaction distance **(Figure 7I)**. Circular dichroism results showed that Sin B increased the α-helical content in the PXR-LBD region **(Figure 7J)**. Finally, we compared the structural changes after binding of Sin B to PXR and found that Sin B induced an upward shift in the AF-2 region, which locked the LBD-binding pocket, and PXR-LBD exhibited a closed conformation** (Figure 7K-L)**. Thus, Sin B binds to PXR and induces conformational changes in the PXR-LBD domain.

### 3.8 Sin B loses its hepatoprotective effects in *Pxr*-knockdown mice

**Figure 8:**
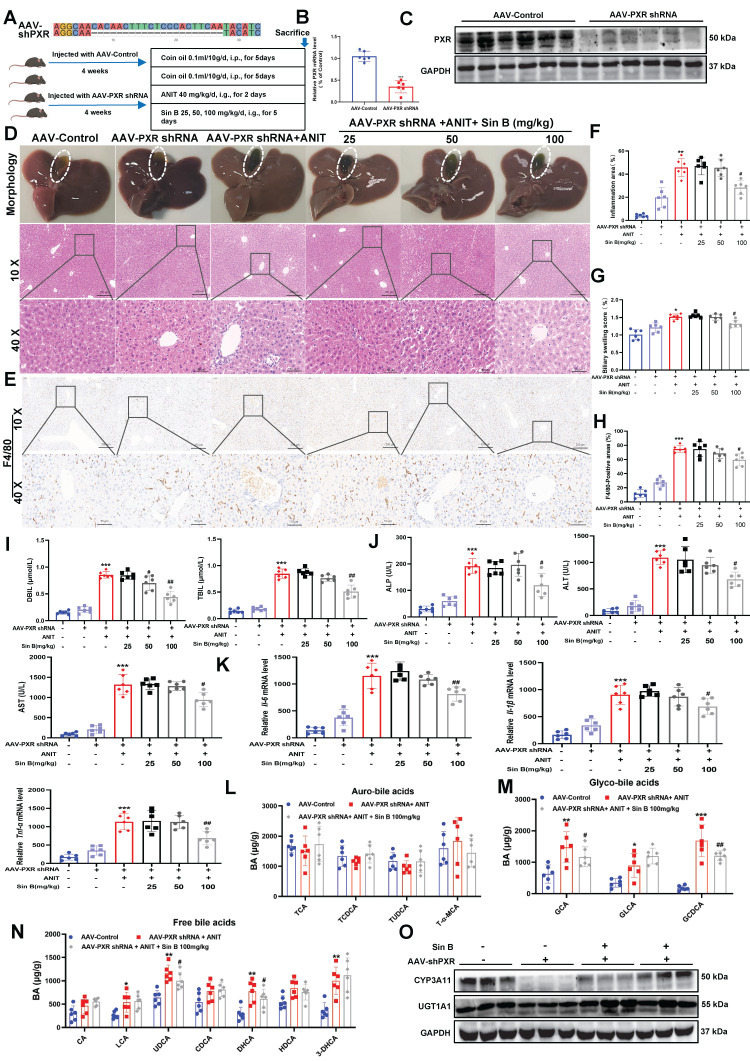
** Sin B attenuates protective effects in AAV-PXR shRNA mice.** (A) *Pxr* knockout sequence and overview of ANIT-induced CLI model creation and Sin B treatments in AAV-PXR shRNA mice. (B-C) PXR mRNA and protein expression analyzed by RT-qPCR and western blot, respectively. (D) Liver morphology and H&E staining for each group. (E-F) Inflammation area and evaluation of bile duct swelling. (G-H) IHC evaluated the F4/80 expression and statistics of liver tissue. (I-J) Serum DBIL, TBIL, ALP, ALT, and AST levels in serum. (K) Expression of the inflammatory factors *Il-6*, *Il-1β* and *Tnf-α* in liver tissues as quantified by RT-qPCR. (L-N) LC-MS/MS for the identification of the types and abundance of free BAs, tauro-BAs and glyco-BAs in each group of mice. (O) The western blot for the CYP3A11 and UGT1A1 expression level measurement. All experiments were repeated six times, and *t*-tests were used to determine statistical significance when comparing AAV-PXR shRNA versus AAV-PXR shRNA+ANIT (^*^P < 0.05, ^**^P < 0.01) and AAV-PXR shRNA + ANIT versus AAV-PXR shRNA+ANIT+Sin B (^#^*P* < 0.05).

Many studies have reported that *Pxr* deletion causes BA metabolic disorders in mice. To verify the specificity of Sin B for PXR *in vivo*, we generated *Pxr*-knockdown mice using an adeno-associated virus (AAV-PXR shRNA), and induced CLI using ANIT **(Figure 8A)**. Both PXR mRNA and protein levels were reduced by approximately 68% in the AAV-PXR shRNA group compared to those in the control group **(Figure 8B-C)**. We examined the expression of other nuclear receptors and found that the protein and mRNA expression levels of FXR and CAR in the AAV-PXR shRNA group were unaffected compared to those in the control group. Compared to the model group, the administration of different doses of Sin B failed to improve the protein and mRNA expression of FXR and CAR **(Figure S4)**. The gallbladders of the AAV-PXR shRNA group were dark brown with hemorrhagic spots and adhesions on the liver surface. After the administration of either 25 or 50 mg/kg Sin B, obvious hemorrhagic spots were still visible in the liver, and the gallbladders remained dark brown. Only the highest dose (100 mg/kg) showed mild improvement **(Figure 8D)**. However, liver tissue damage persisted compared with that in the AAV control group. In addition, inflammation and bile duct swelling persisted compared to those in the ANIT group **(Figure 8E-F)**. IHC staining revealed elevated F4/80 expression in AAV-PXR shRNA and ANIT groups. The highest dose of Sin B slightly decreased F4/80 expression; however, this effect was not statistically significant **(Figure 8G-H)**. Serum ALP, ALT, AST, DBIL, and TBIL levels were reduced by Sin B treatment, but only at the highest dose **(Figure 8I-J)**. Similarly, *Il-1β*, *Il-6*, and *Tnf-α* mRNA levels were significantly reduced in the high-dose group, but not to the levels in the control group **(Figure 8K)**. The ANIT group showed higher GCA and GCDCA levels, which decreased marginally after Sin B treatment **(Figure 8L-N)**. Western blotting showed that Sin B partially restored CYP3A11 expression in the AAV-PXR shRNA group but had no effect on UGT1A1 expression **(Figure 8O)**. Thus, the protective effects of Sin B against ANIT-induced CLI were inhibited in AAV-PXR shRNA mice, further confirming that Sin B targets PXR to alleviate CLI inflammatory responses and promote BA metabolism.

### 3.9 Sin B accelerates BAs metabolism by promoting the autophagic lysosome formation in a PXR-dependent manner

**Figure 9:**
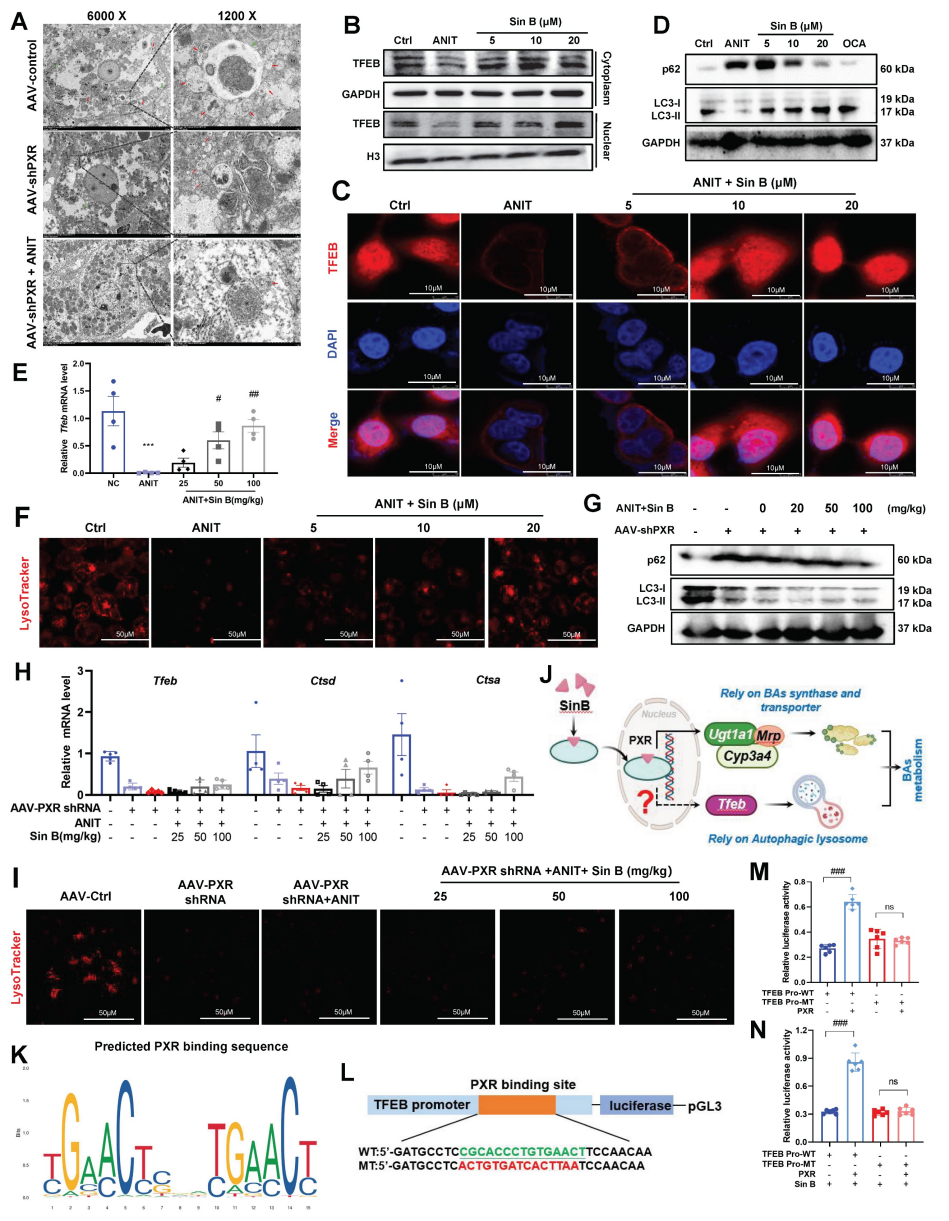
** Sin B activates PXR in the nucleus and PXR binds to the TFEB promoter to regulate TFEB expression.** (A) Morphological changes in autophagosomes, lysosomes, and autophagosomes in AAV-control, AAV-shPXR, and AAV-shPXR+ANIT groups as analyzed by TEM. (B)Western blot analysis of TFEB protein expression in the nucleus and cytoplasm. (C) Localization and protein expression of TFEB as analyzed by confocal microscopy. Scale bar, 20 mm. (D) The western blot for the P62 and LC3 expression level measurement. (E) Expression of the *Tfeb* in liver tissues as quantified by RT-qPCR. (F) Representative images of LysoTracker staining in WRL68 cells and quantification of fluorescence intensity and expressed as fold change relative to control group. Scale bar, 50 μm. (G) The western blot for the P62 and LC3 expression level measurement. (H) Expression of the *Tfeb, Ctsd and Ctsa* in liver tissues as quantified by RT-qPCR. (I) Representative images of LysoTracker staining in WRL68 cells and quantification of fluorescence intensity and expressed as fold change relative to control group. Scale bar, 50 μm. (J) Schematic diagram of Sin B targeted PXR regulation of BAs metabolism. (K) Transcription factor PXR binding sequence predicted in the JASPAR database. (L)Schematic diagram of the luciferase reporter wild-type construct (WT) containing the TFEB promoter and the mutant TFEB construct (MT) containing the TFEB promoter with the mutant PXR binding sites. (M) The binding of PXR to the TFEB promoter revealed by dual luciferase reporter assay. (N) Effect of Sin B combination on the binding between PXR and TFEB promoter revealed by the dual luciferase reporter assay.

To study the hepatoprotective mechanisms of Sin B beyond BA metabolism, we assessed organelle morphology in the AAV control, AAV-PXR shRNA, and AAV-PXR shRNA+ANIT groups using TEM. As shown in **Figure 9A**, abundant autophagolysosomes were observed along with normal autophagosomes. In contrast, the AAV-PXR shRNA group exhibited fewer autophagosomes, and the AAV-PXR shRNA +ANIT group showed an almost complete absence of autophagolysosomes. Western blotting and IF assays revealed that TFEB levels in the nucleus were increased after Sin B treatment **(Figure 9B-C)**. Furthermore, Sin B administration increased the degradation of P62 and formation of LC3-II in ANIT-induced CLI mice **(Figure 9D)**, indicating that Sin B elevated hepatic autophagic flux. RT-PCR analysis further verified that Sin B treatment elevated the level of the autophagy-regulated gene *Tfeb* **(Figure 9E)**. Sin B treatment dramatically enhanced lysosomal function, as indicated by LysoTracker staining **(Figure 9F)**. These data demonstrate that Sin B activates the autophagic lysosomal pathway in the liver of CLI mice. Importantly, in hepatocyte-specific *Pxr*-knockout mice, Sin B did not significantly affect hepatic autophagic activity, as measured by the protein abundance of P62 and LC3-II **(Figure 9G)**, the mRNA levels of *Tfeb, Ctsa* and *Ctsd* **(Figure 9H)**, or lysosomal function, as indicated by LysoTracker staining **(Figure 9I)**. However, the specific mechanism through which PXR regulates lysosomal autophagy remains unclear **(Figure 9J)**.

Moreover, the binding site (-CGCACCCTGTGAACT) of the TFEB promoter sequence for PXR was predicted using the bioinformatics tools PROMO and JASPAR **(Figure 9K)**. The TFEB core promoter reporter gene plasmids and mutant plasmids were constructed for luciferase activity assay **(Figure 9L)**. As shown in **Figure 9M**, the luciferase activity of the TFEB promoter in cells overexpressing PXR was significantly higher than that in the control group (wild-type, WT). Mutation of the PXR-binding site significantly reduced the luciferase activity to a level lower than that of the WT group. These results indicate that PXR is involved in the transcription of TFEB and that PXR binds to the promoter region of TFEB. Therefore, PXR may directly bind to the TFEB promoter region to enhance TFEB transcription. As shown in **Figure 9N**, the interaction between the TFEB promoter and PXR increased luciferase activity by approximately 30%, and Sin B treatment enhanced the PXR-induced luciferase activity by approximately 50%. These findings indicate that Sin B enhances the interaction between PXR and the TFEB promoter. Taken together, these results indicate that Sin B targets PXR to activate TFEB and promote autophagic lysosome formation**.**

### 3.10 Sin B loses its hepatoprotective effects in *Tfeb* -knockdown mice

**Figure 10:**
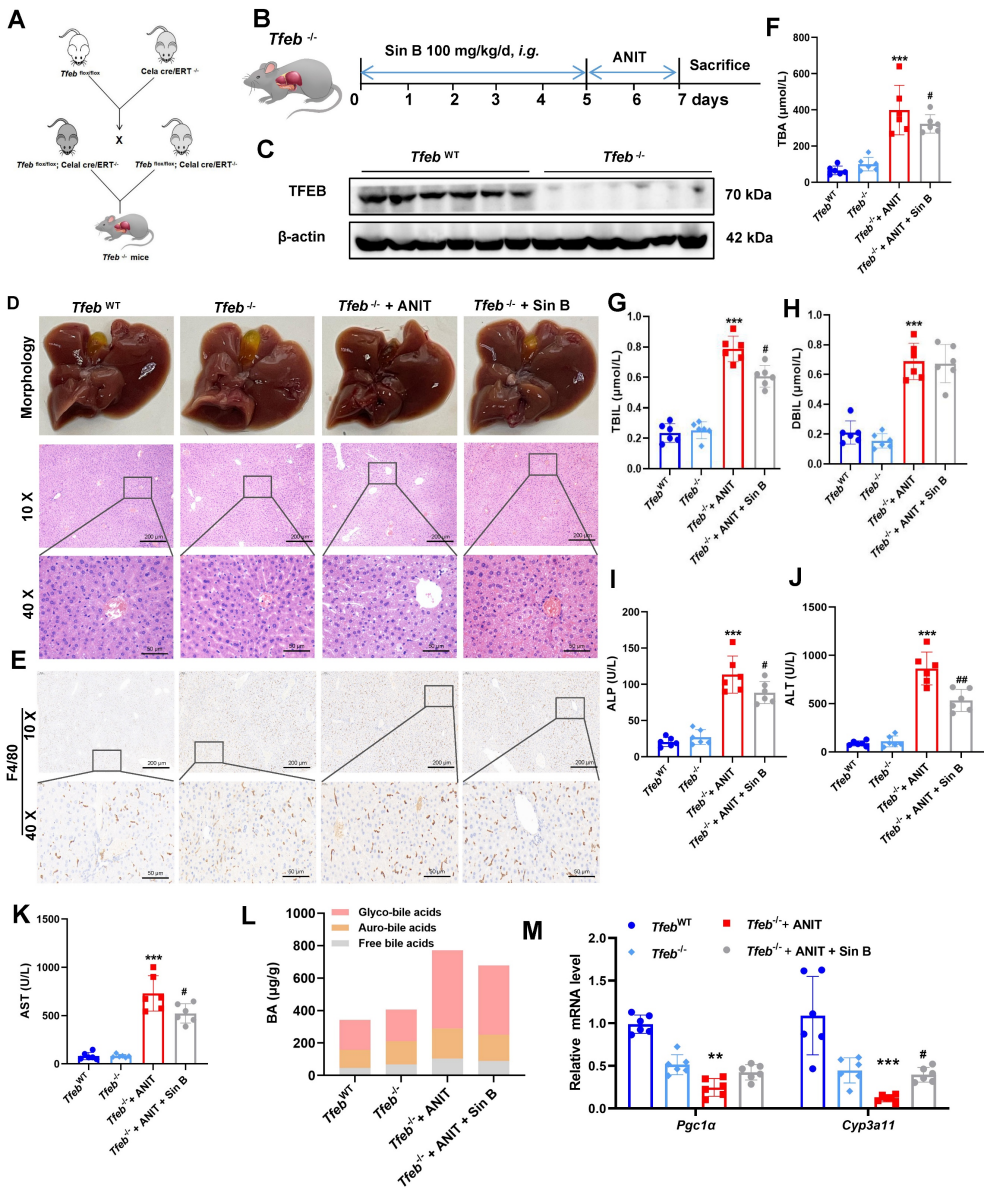
** Knockout of hepatic *Tfeb* blocks the alleviation effects of Sin B on CLI in mice.** (A) Creation of *Tfeb*^-/-^ mice. (B)*Tfeb*^-/-^ mouse CLI model construction and flow chart for Sin B administration. (C) Western blot of TFEB protein expression. (D) Liver morphology and H&E staining for each group. (E) Immunohistochemistry evaluation of F4/80 expression. (F-K) Serum TBA, TBIL, DBIL, ALP, ALT, and AST levels in serum. (L) BAs metabolomics used to assess the abundance of free BAs, glyco-BAs, and tauro-BAs in liver tissues from each group. (M) RT-qPCR analysis of mRNA expression for *Pgc1α* (downstream of TFEB) and *Cyp3a11* (downstream of PXR). All experiments were repeated six times, and *t*-tests were used to determine statistical significance when comparing AAV-PXR shRNA versus AAV-PXR shRNA+ANIT (^*^P < 0.05, ^**^P < 0.01) and AAV-PXR shRNA + ANIT versus AAV-PXR shRNA+ANIT+Sin B (^#^*P* < 0.05). *Tfeb*^-/-^ compaired with *Tfeb*^-/-^+ANIT (^*^P <0.05, ^**^P <0.01, ^***^P <0.001); *Tfeb*^-/-^ + ANIT compaired with *Tfeb*^-/-^ +ANIT+Sin B (^#^*P* < 0.05, ^##^*P* < 0.01).

The effects of Sin B on TFEB were explored in *Tfeb*^-/-^ mice, and an ANIT-induced CLI model was also constructed in *Tfeb*^-/-^ mice **(Figure 10A, B)**. Western blotting confirmed that TFEB was effectively eliminated from the livers of *Tfeb*^-/-^ mice** (Figure 10C)**. The liver tissues of mice in the *Tfeb*^-/-^ group were enlarged and to a lesser degree in the Sin B group. H&E staining revealed significantly more inflammatory infiltration in the *Tfeb*^-/-^ group than that in the Sin B group **(Figure 10D)**. Additionally, IHC staining analysis revealed higher F4/80 expression in the *Tfeb*^-/-^ mice compared to the Sin B group **(Figure 10E)**. These results suggested that Sin B alleviates ANIT-induced inflammatory damage in *Tfeb*^-/-^ mice. TBA, TBIL, DBIL, ALP, ALT, and AST levels were elevated in the CLI model compared with the *Tfeb*^WT^ group as well as the *Tfeb*^-/-^+ANIT group. Administration of Sin B significantly reduced these levels, although the absolute reductions were modest **(Figure 10F-K)**. In *Tfeb*^-/-^ mice, free BAs and glycine-bound BAs decreased after Sin B treatment compared to the ANIT group; the GUDCA content also significantly decreased, with no notable effect on other BAs** (Figure 10L)**. The expression levels of* Cyp3a11* and *Pgc1α* (as measured by RT-qPCR) were decreased in the *Tfeb*^-/-^ group but were enhanced after Sin B administration **(Figure 10M)**. This suggests that Sin B is only involved in the regulation of certain BAs in *Tfeb*^-/-^ mice, with little effect on most BAs (possibly due to *Tfeb* deletion). Taken together, our data demonstrates that activation of the PXR-TFEB signaling pathway plays a central role in the hepatoprotective effects of Sin B.

## 4. Discussion

*Schisandra chinensis*, a traditional herb used across East Asia, contains bioactive lignans that are known for their antioxidant, anti-inflammatory, and hepatoprotective properties, particularly against CLI 30-33. According to the research report by Liang et al., we have determined the dosage of Sin B in the *in vivo* model induced by ANIT 11. Among them, Sin B demonstrated the highest efficacy. It significantly reduced ALT and AST levels, attenuated hepatocyte apoptosis and fibrosis, and bound directly to PXR with high affinity, as confirmed by ITC and SPR assays 34, 35.

A key innovative finding is that Sin B induces conformational changes in the PXR LBD, stabilizing residues S106, G144, and the AF-2 helix, which facilitates coactivator recruitment and enhances receptor activity 36. This structural insight provides a novel basis for future drug design and optimization. The precise elucidation of the binding mode of Sin B and the induced conformational changes in PXR offers a template for structure-based drug design. Future studies should focus on modifying the Sin B scaffold to enhance its binding affinity or selectivity for PXR. Furthermore, understanding how these specific interactions trigger distinct conformational shifts could guide the development of novel PXR modulators that favor beneficial metabolic pathways while minimizing untoward effects and potentially overcoming the inherent challenges of targeting this promiscuous nuclear receptor.

Mechanistically, Sin B activates PXR to regulate BA homeostasis by inhibiting the synthesis enzymes Cyp7a1, Cyp27a1, Cyp7b1, and Cyp8b1 (Figure S5), and upregulating the detoxifying enzymes CYP3A4 (in humans) or CYP3A11 (in mice) and the transporters Ugt1a1. Using *Pxr*-knockdown mice, we validated the central role of PXR in mediating the protective effects of Sin B against CLI. Furthermore, Sin B enhanced TFEB-mediated autolysosome formation, promoting BA clearance. However, the partial efficacy observed in *Tfeb*^⁻⁄⁻^ mice indicates that Sin B also acts through TFEB-independent pathways, such as direct PXR-mediated upregulation of metabolic enzymes and transporters.

Notably, PXR activation has systemic implications, representing a quintessential “double-edged sword”. Although beneficial for CLI, the potent induction of CYP3A and UGT1A1 by Sin B raises significant risks for drug-drug interactions (DDIs), potentially altering the metabolism of co-administered drugs 37, thus necessitating careful consideration of its clinical translatability. For instance, CYP3A4 induction by Sin B indicates that it can significantly accelerate the clearance of concomitant medications that are substrates of this enzyme, potentially compromising their therapeutic efficacy. Preclinical DDI studies and eventual clinical pharmacokinetic evaluations are necessary to define its interaction profile and identify potential contraindications. Additionally, PXR modulates glycolipid metabolism and immune function 38-40, necessitating careful long-term benefit-risk evaluation. Systemic activation of PXR by Sin B could lead to dyslipidemia or altered glucose homeostasis, as suggested by studies using other PXR agonists 38. Therefore, future chronic toxicology studies using relevant models should specifically monitor these metabolic parameters to ensure a comprehensive safety profile.

Our comprehensive analysis indicates that the multi-target antioxidant properties of Sin B are superior to the existing therapies OCA and UDCA. Although OCA has certain efficacy, it causes itching and reduces high-density lipoprotein cholesterol levels. While UDCA, as a first-line treatment drug, is ineffective for up to 40% of patients. Thus, Sin B represents a promising multitarget candidate, although its clinical translation requires further mechanistic elucidation and safety validation. This study has several limitations. The stable and reproducible WRL68 cell line may not fully recapitulate primary human hepatocyte physiology. Future studies should employ cell-type-specific models, such as HSC-specific knockouts or advanced co-cultures, to precisely delineate anti-fibrotic mechanisms. Further long-term studies and detailed DDI profiling are essential to fully establish the therapeutic potential and safety of Sin B as a PXR-targeting agent for cholestatic liver disease.

## Supplementary Material

Supplementary figures.

## Figures and Tables

**Table 1:** Sequence (5'-3') of primers used for real time quantitative PCR.

Gene	Forward (5'→3')	Reverse (5'→3')
*Il-1β* (WRL68 cell)	TGCCACCTTTTGACAGTGATG	TGTGCTGCTGCGAGATTTGA
*Il-6* (WRL68 cell)	TCCTTCCTACCCCAATTTCCA	GCACTAGGTTTGCCGAGTAGA
*Tnf-α* (WRL68 cell)	TGCTTGTGTCTGTCTTGCGT	CCCGTGAATCCACCATGTCT
*Gapdh* (WRL68 cell)	CGGCCGCATCTTCTTGTG	GTGACCAGGCGCCCAATAC
*Il-1β* (Mouse)	TGCCACCTTTTGACAGTGATG	TGTGCTGCTGCGAGATTTGA
*Il-6* (Mouse)	TCCTTCCTACCCCAATTTCCA	GCACTAGGTTTGCCGAGTAGA
*Tnf-α* (Mouse)	TGCTTGTGTCTGTCTTGCGT	CCCGTGAATCCACCATGTCT
*Gapdh* (Mouse)	GGAGAAACCTGCCAAGTATGA	CCTGTTGCTGTAGCCGTATT
*Mrp3* (Mouse)	CTGGGTCCCCTGCATCTAC	GCCGTCTTGAGCCTGGATAAC
*Mrp4* (Mouse)	CATCGCGGTAACCGTCCTC	CCGCAGTTTTACTCCGCAG
*Oatp1b1* (Mouse)	GCACTGCGATGGATTCAGGAT	AGCTTTGGTCGGTGTAGCTTG
*Ctsa* (Mouse)	GCTTCCGGTCTTTGACAACCT	CACCAAGCATTAGTTCTCCTCC
*Ctsd* (Mouse)	TGCCACTTAAGCGGAGATTT	AGGTAGTTGGAAGGGGCTGT
*Cyp3a11* (Mouse)	GACAAACAAGCAGGGATGGAC	CCAAGCTGATTGCTAGGAGCA
*Ugt1a1* (Mouse)	GTCATCCAAAGACTCGGGCA	GACATTCAGGGTCACCCCAG
*Fxr* (Mouse)	GCTTGATGTGCTACAAAAGCTG	CGTGGTGATGGTTGAATGTCC
*Car* (Mouse)	CTCCCCTGGTGAGGATCATC	GACCGAGAGTTGGGTAGAGGT

**Table 2:** Data collection and refinement statistics.

Crystals	PXR Apo
	Data collection
Space group	*P* 21 21 21
a, b, c (Å)	87.23, 88.41, 106.05
α, β, γ (°)	90.00, 90.00, 90.00
Resolution (Å)	22.9 - 3.0 (3.1 - 3.0) ^a^
R_merge_^b^	0.198 (0.380)
I/σ_(I)_	4.3 (2.6)
Completeness (%)	92.40 (100.00)
Redundancy	4.1(4.8)
	Refinement
Resolution (Å)	22.9 - 3.0 (3.1 - 3.0)
No. reflections	15618 (1664)
R_work_^c^/R_free_^d^	0.3062/0.3504
No. atoms	4707
Protein	581
Ligand	
Water molecules	18
	B-factors (Å^ 2^)
Protein	54.7
Ligand	
Water molecules	10.3
Bond lengths (Å)	0.014
Bond angles (º)	1.89
Ramachandran favored (%)	89.56
allowed	9.20
Outliers (%)	1.24

^a^Values in parentheses are for the highest-resolution shell. ^b^R_merge_ =Σ (I - < I >) |/σ(I), where I is the observed intensity. ^c^R*_work_* = Σ_hkl_ ||Fo| - |Fc||/ Σ_hkl_ |Fo|, calculated from working data set. ^d^R*_free_* was calculated from 5.0% of the data, selected at random and not included in the refinement analysis.

## Abbreviations

ALT: alanine aminotransferase
AST: aspartate aminotransferase
ALP: alkaline phosphatase
ANIT: α-naphthylisothiocyanate
BAs: bile acids
CLI: Cholestatic liver injury
EdU: 5-Ethynyl-2'-deoxyuridine
CETSA: cellular thermal shift assay
DDB: Bifendate
DARTS: drug affinity responsive target stability
DBIL: direct bilirubin
DHCA: dehydrocholic acid
3-DHCA: 3-dehydrocholic acid
FXR: foresaid X receptor
GCDCA: glycochedeoxycholic acid
ITC: isothermal titration calorimetry
MRP2: multi-drug resistance associated protein 2
OCA: obetcholic acid
PXR: Pregnane X receptor
Sin A: schisandrin A
Sin B: schisandrin B
Sol A: schisandrol A
Sol B: schisandrol B
Shin A: schisantherin A
Shin B: schisantherin B
Son L: schisanhenol
SPR: surface plasmon resonance
TFEB: transcription factor EB
TBA: total bile acid
TBIL: total bilirubin
TCA: taurocholic acid
T-α-MCA: taurine-amidated α-MCA
GCA: glycocholic acid
UDCA: ursodeoxycholic acid
OATP1B1: organic anion transporting polypeptide 1B1
