# Supplementary material for: Schisandrin B Targets PXR to Enhance Bile Acid Metabolism and Alleviate ANIT-Induced Cholestatic Liver Injury via Dual Pathways
Source: Int J Biol Sci. 2026 Jan 1;22(1):387–409. doi: 10.7150/ijbs.121475 (PMC12681940; doi:10.7150/ijbs.121475)
Supplement: Supplementary file 1 — Supplementary figures. [file ijbsv22p0387s1.pdf]

Supplementary materials

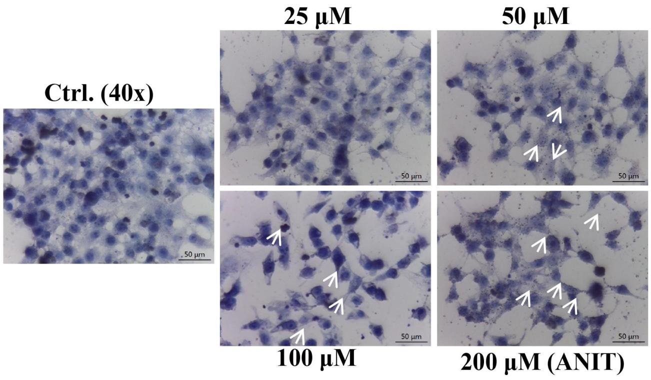

Supplementary Figure 1. ANIT gamaged WRL68 cells morphology. WRL68 cells were treated with 25, 50, 100 and 200 µM ANIT for 24 h, and the cells showed internal fluid drain, nuclear exposure, and rounded morphology and tended to die (Scare bar=50 µM).

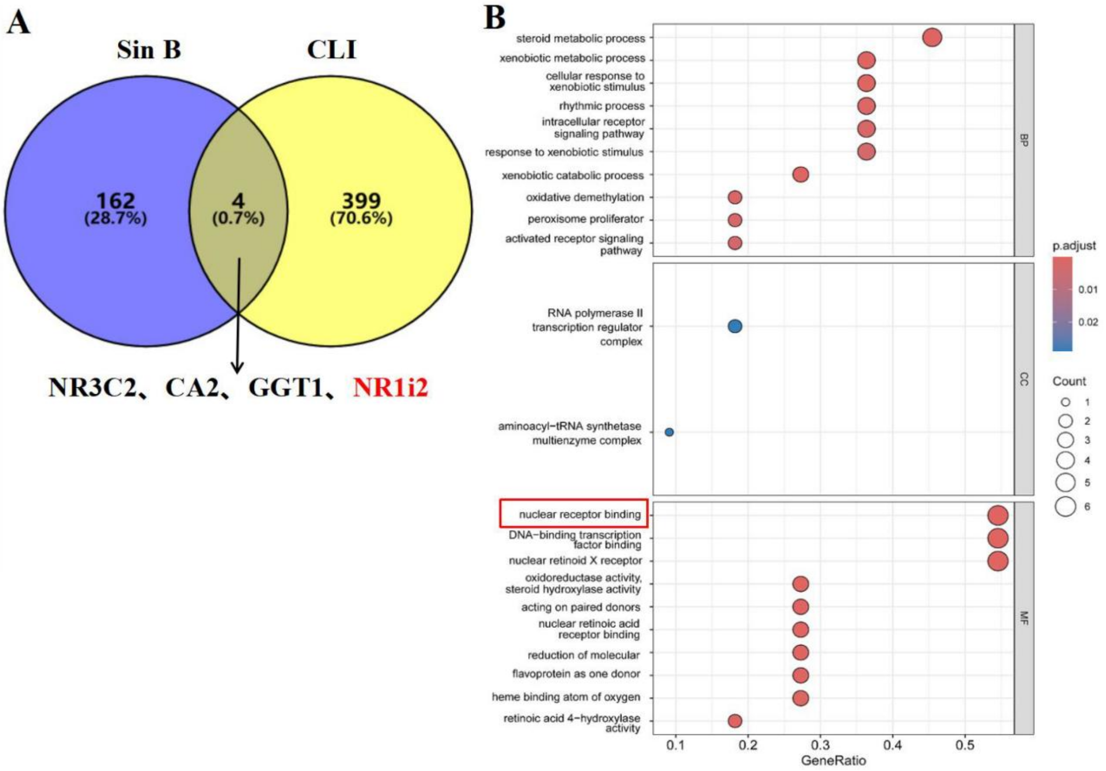

Supplementary Figure 2. Network pharmacological analysis of anti-CLI effects of Sin B. (A) Four targets asssociated with CLI intersected with Sin B targets. Purple represents the number of potential Sin B targets, and the yellow represents the number of potential CLI targets. (B) Sin B participation in KEGG pathway that regulate CLI.

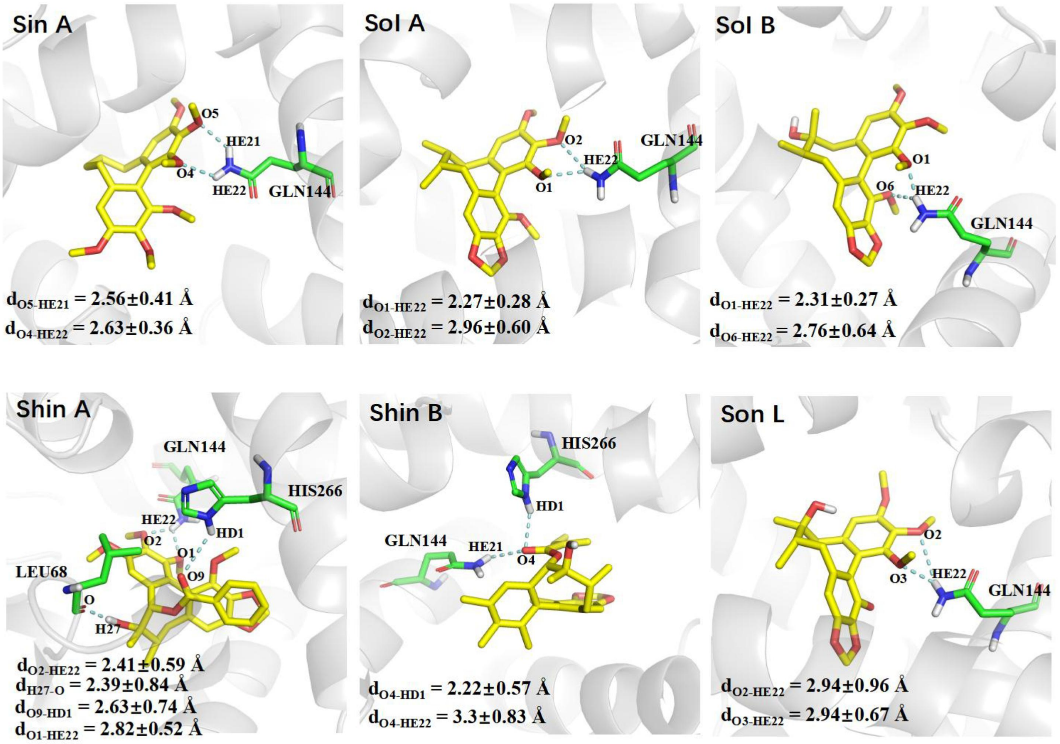

Supplementary Figure 3. Molecular docking analysis of lignans with PXR protein. Molecular docking analysis of key interactions sites and distances for Sin A, SoI A, SoI B, Shin A, Shin B and Son L with the PXR protein.

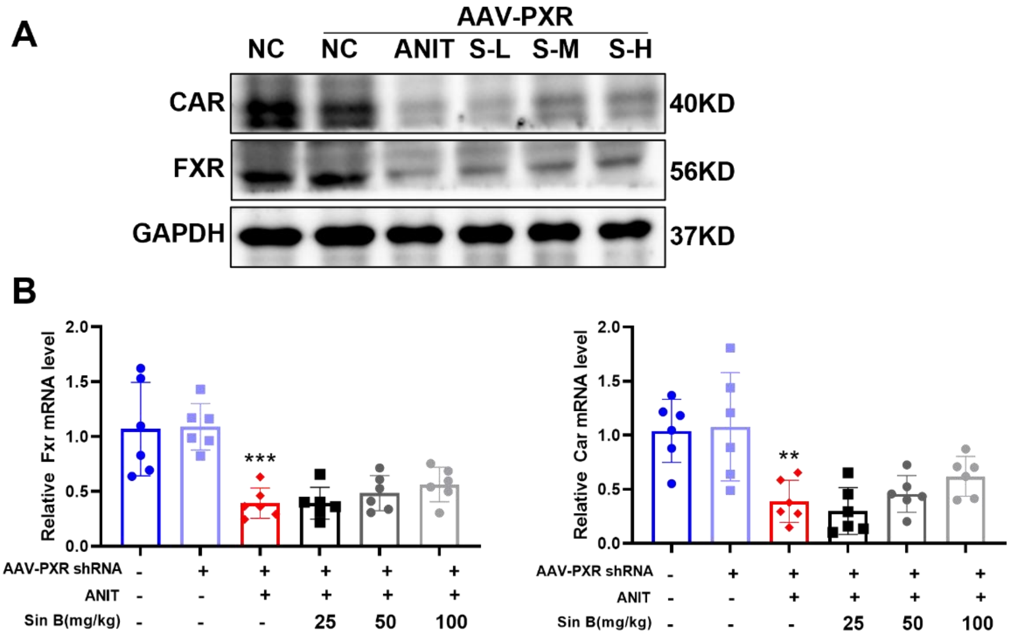

Supplementary Figure 4. Effect of Sin B on FXR and CAR expression in AAV-PXR shRNA mice. (A) The protein expression levels of FXR and CAR were analyzed by western blot. (B)The mRNA expression levels of *Fxr* and *Car*, were analyzed by RT-qPCR.

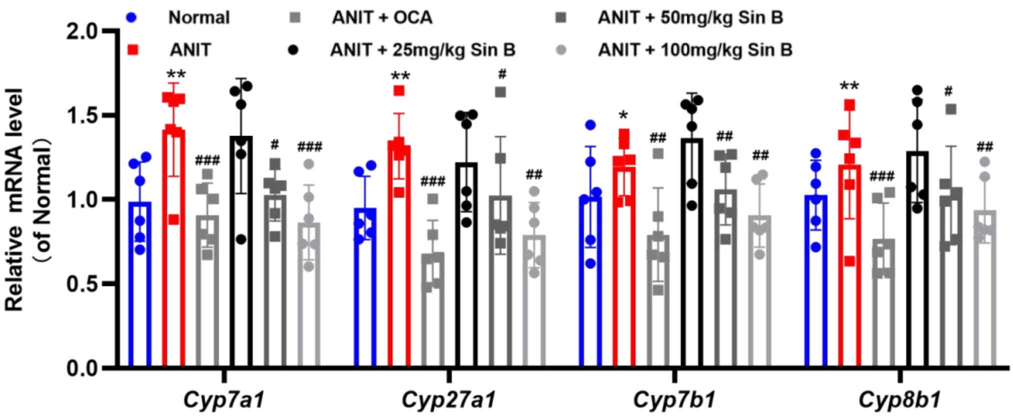

Supplementary Figure 5. Effect of Sin B on bile acid synthase expression *in vivo*. The mRNA expression levels of bile acid synthase *Cyp7a1*, *Cyp27a1*, *Cyp7b1* and *Cyp8b1* were analyzed by RT-qPCR.
